# Towards an anthropomorphic MRI phantom mimicking static and dynamic B_0_ field variations in the human cervical spinal cord

**DOI:** 10.1007/s10334-026-01349-4

**Published:** 2026-04-15

**Authors:** Laura Beghini, Brunnhilde M. A.-S. Ponsi, Kamilla Refsholt, Annelen Dogger Schmidt, Virginie Callot, S. Johanna Vannesjo

**Affiliations:** 1 Department of Physics, Norwegian University of Science and Technology, Trondheim, Norway; 2 CRCI2NA, INSERM UMR 1307, Nantes, France; 3 Nuclear Medicine Department, University Hospital, Nantes, France; 4 Aix-Marseille Université, CNRS, CRMBM UMR 7339, Marseille, France; 5 AP-HM, Hopital de La Timone, Pôle d’imagerie Médicale, CEMEREM, Marseille, France

**Keywords:** Spinal cord, Phantom, B_0_ field inhomogeneity, Artifacts, Breathing

## Abstract

**Objective:**

Spinal cord MRI is heavily affected by static and dynamic B_0_ field variations. The aim of this study was to develop an anthropomorphic cervical spine phantom that replicates these.

**Materials and Methods:**

The phantom consisted of a 3D-printed head-to-thorax shell containing 3D-printed vertebrae (C1–T1) in a water-based liquid solution. An external respiration system mimicked breathing-induced field fluctuations by moving metal staples over the phantom. The accuracy of the reproduced fields was evaluated at 7 T by field maps and multi-echo GREs acquired in the phantom and in four subjects.

**Results:**

Fourier-based field simulations confirmed the vertebrae to be the main contributors to the local static field in the spinal canal, providing a rationale for the phantom design. The 3D-printed vertebrae accurately reproduced the spatial field pattern encountered in vivo, but with one-third of the intensity due to lower susceptibility differences. The respiration system produced spatially and temporally similar dynamic field fluctuations as in vivo.

**Discussion:**

The phantom was designed to be low-cost, modular and reproducible, while also being non-toxic and free from biological hazards. It may serve as a useful tool for development and testing of correction strategies to address the persistent challenge of B_0_ field variations in spinal cord MRI.

**Supplementary Information:**

The online version contains supplementary material available at https://doi.org/10.1007/s10334-026-01349-4.

## Introduction

Innovation in MRI requires frequent testing of protocols and sequences. First stage testing is mostly done on homogeneous phantoms. These phantoms, however, fail to represent key challenges encountered in vivo, related to human anatomy and physiology. Phantoms reproducing these specific challenges can aid the testing process and thereby speed up development. One such challenge is the high sensitivity of T_2_*-weighted sequences to B_0_ field variations in the form of spatial inhomogeneity and temporal instability.

Macroscopic B_0_ field variations in space and in time can dephase signal and disrupt spatial encoding, resulting in artifacts such as signal loss, geometric distortion and ghosting. Local field gradients due to static B_0_ field inhomogeneity, particularly in the through-slice direction, dephase the signal, thereby causing signal loss which is particularly severe in T_2_*-weighted images with long echo times. In-plane, the local static field gradients perturb the spatial encoding performed by the gradient system of the scanner, leading to geometric distortion in the images. The distortion can affect both T_2_*-weighted imaging and other sequences, increasing with low acquisition bandwidth and long readouts. Finally, B_0_ field fluctuations over time introduce phase variations in the acquired signal, and typically cause ghosting in T_2_*-weighted multi-shot acquisitions, especially at long TE.

Spinal cord imaging, in particular at 7 T, is heavily affected by both static and dynamic B_0_ field variations [1, 2]. Two main components contributing to static field inhomogeneity can be identified within the spinal cord. One is a long-range component, often referred to as background field, that originates from the susceptibility difference between air and tissues, resulting in a smoothly varying field offset throughout the spinal cord. The other component manifests on a smaller spatial scale and is localized around interfaces between vertebrae [3–7], intervertebral discs, and soft tissue [8]. This local component exhibits a characteristic alternating pattern [1, 9] that causes signal drop-out and geometric distortion around each intervertebral junction in T_2_*-weighted images. Dynamic B_0_ field fluctuations are mainly caused by variations of air volume in the lungs during the breathing cycle, and yield ghosting artifacts and signal loss in T_2_*-weighted images [2, 10]. Various techniques have been proposed to minimize these effects, including slice-wise shimming [11–14] to reduce the effect of static field inhomogeneity, as well as real-time shimming methods [11, 15] and retrospective corrections [16, 17] to compensate for the breathing-induced field fluctuations. Nevertheless, substantial artifacts persist, especially at 7 T.

In this context, an anthropomorphic phantom of the human spine that can reproduce the static and dynamic B_0_ fields encountered in vivo could facilitate the development of advanced acquisition and correction techniques. Such a phantom would reduce the need for in vivo scanning during the early development phase, while also permitting longer testing sessions than are feasible in vivo. Additionally, it would enable the reliable reproduction of experimental conditions and facilitate comparison between different correction techniques.

While the different components of static and dynamic B_0_ field inhomogeneity have been individually reproduced in phantoms, no previous work has combined all of them.

Lopez-Rios et al. [18] proposed an anthropomorphic-shaped phantom of the human head and neck with a uniform filling for coil quality assurance. Though the phantom could potentially reproduce the background component of the static field, its B_0_ field characteristics were not evaluated. Phantoms of vertebrae fabricated from artificial materials [19–21], including nerves, muscles, bone marrow, and intervertebral discs [19], have also been proposed in order to provide tissue contrasts similar to the human body in MR and CT. However, these phantoms were developed for image-guided interventions, without an external shell to allow access, and the susceptibility characteristics of the used materials along with the resulting B_0_ field distribution were not investigated.

To mimic local static and dynamic B_0_ fields, De Tillieux et al. [22] introduced a phantom equipped with a section of an ex vivo human spine, including connective tissue, vertebral bones and spinal cord, positioned in a plastic box containing a conservation liquid and two mechanically resistant “punch-balloon” lungs that were inflated and deflated by an automatic system to generate periodic field fluctuations with a consistent waveform. This design, however, presents some practical drawbacks. Due to its size, the phantom can only be scanned using the body coil for both excitation and reception (at 3 T). Furthermore, the phantom cannot be consistently replicated, due to the use of ex vivo material. It also introduces a biological hazard, requiring the presence of a toxic additive (phenol) in the phantom solution, which limits the possibility for intra-session modifications. Alonso-Ortiz et al. [11] demonstrated that periodic field fluctuations can alternatively be induced by moving iron staples near the imaging target. However, this was not combined with a phantom reproducing the static field inhomogeneity. Furthermore, the intensity and time course of the field fluctuations were not compared to the breathing-induced field fluctuations encountered in vivo.

The aim of this study was to construct a phantom of the human cervical spine that can mimic both the static (background and local) and dynamic B₀ field variations encountered in vivo. The phantom was designed to be low-cost, reproducible and adaptable to emulate specific subjects. The static B_0_ fields were achieved by inserting 3D-printed vertebrae into a 3D-printed anthropomorphic phantom shell filled with a non-toxic uniform solution [20, 21, 23, 24]. This phantom design is based on the assumption that the vertebrae [3–5] are the primary contributors to the local static fields, while surrounding soft tissues have a comparatively lower impact on the field distribution [8]. We verified this assumption by comparing field simulations with acquired field maps. The dynamic field fluctuations were generated by an external, removable respiration system that could automatically move iron staples near the phantom’s thorax based on breathing patterns recorded in vivo. The resulting static and dynamic B_0_ field perturbations were quantified and compared with in-vivo measurements to evaluate their accuracy. Additionally, the effect of the B_0_ field perturbations on T_2_*-weighted GRE sequences was visually assessed. Preliminary results of this work have previously been published in conference abstracts [25–27].

## Materials and methods

### Subjects

Four healthy volunteers (2 females, mean age (range): 28.5 (23–34) years old, mean body mass index (BMI) (range): 21.4 (19.1–24.2) kg/m^2^) participated in this study. Subject 4 was randomly selected as the template for the phantom vertebrae and breathing pattern. The remaining subjects provided contextual reference.

### Phantom design

**Fig. 1:**
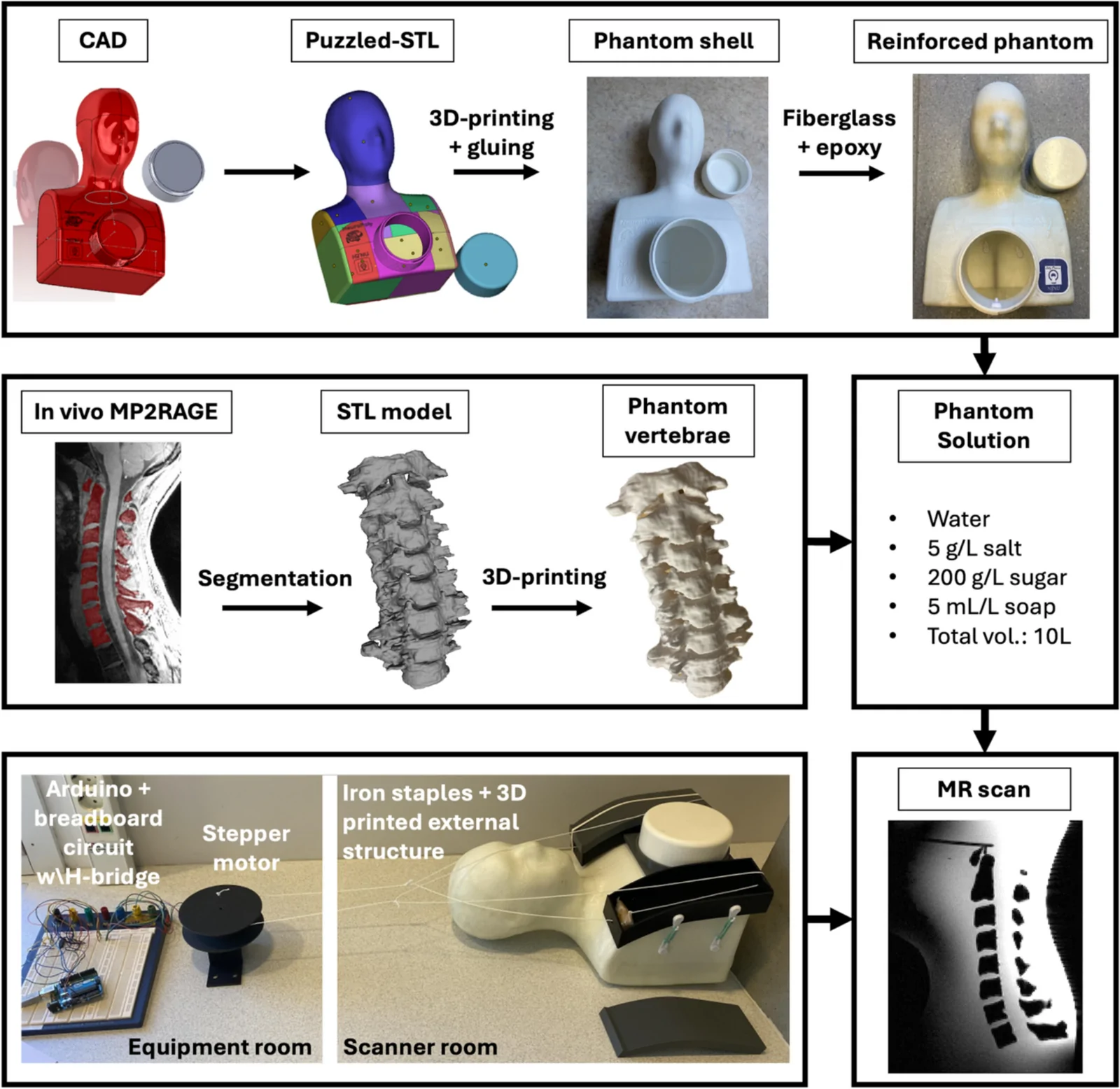
Schematic workflow for constructing the anthropomorphic phantom. More details regarding the vertebral structural stability and the respiratory system are provided in Figures S1 and S3

The main considerations of the phantom design are described in this section, whereas specific details are given in the Supplementary Materials. The phantom consisted of an external shell containing a water-based liquid solution, representing soft tissue, and 3D-printed homogeneous vertebrae. The workflow of the phantom construction process is illustrated in Fig. 1. The phantom shell was obtained by modifying an open-source average head and neck model [18] in Solidworks (Dassault Systèmes Solidworks Corporation, 2022). To reduce the cost of 3D-printing and the phantom’s weight, the model was resized to 80% of its dimensions in each direction. As the original model was quite large (corresponding to a large male) [18], the resized model still maintained realistic human proportions (corresponding to a small female). The shell model was divided into pieces as a jigsaw puzzle to optimize the printing process (Fig. 1), before being 3D-printed in nylon and glued together.

The model for the vertebrae was obtained by manually segmenting C1 to T1 on a T1-weighted MP2RAGE sequence [9] (0.7 mm^3^ isotropic resolution, see ‘Data acquisition’ below) acquired on subject 4 (male, age: 23 yo, BMI: 21.4, height: 169 cm). The spine model was then 3D printed using Rigid 10 K, which is known to have negative susceptibility [28] compared to water, as expected for cortical bone compared to soft tissue [3, 5–7]. The 3D-printed spine was positioned inside the phantom with thin threads attached to plastic hooks that were glued to the phantom interior.

The phantom solution consisted of water, 5 g/L of salt to match the conductivity of human tissues [29], 200 g/L to reduce T_2_* (Fig. S2) and to tune the permittivity [29], and 5 mL/L of liquid hand soap to reduce surface tension in order to prevent the formation of small air bubbles around the vertebrae.

### Respiratory system

To mimic breathing-induced field fluctuations in the phantom, we built an automated system to move iron staples [11] over the phantom thorax in a periodic manner. The staples were taped inside two water-filled plastic tubes, which were pulled back and forth over the phantom thorax using threads connected to a rotating stepper motor. To ensure a reproducible pattern of motion, a vest was constructed featuring two distinct enclosed pathways oriented along the foot–head direction. The stepper motor was controlled by an Arduino Uno Rev3 via an SN754410ne H-Bridge (Fig. 1). All electronic components were positioned on a table in the equipment room and the threads were passed through a waveguide located in front of the magnet bore.

The Arduino can control the motor’s rotation direction, speed, and number of steps, and can therefore reproduce arbitrary motion patterns within the limitations of the motor and the circuit. Within a session, the intensity and time evolution of field variations can be controlled by the range of motion of the motor and its programmed motion profile. On a cruder scale, the intensity of field variations can be adjusted between sessions by varying the number of iron staples. During acquisitions mimicking free breathing, 1.5 staples per tube were used. The maximal motor speed was set to 60 RPM, which limited the maximum slope of the motion. The setup was programmed to mimic the trace recorded by a respiratory belt during acquisitions on subject 4, synchronizing the trace with the MR data using the PhysIO toolbox [30]. The resulting breathing pattern was played out on the respiratory system while acquiring the same sequence on the phantom. The respiratory system was manually started at approximately the same time as the sequence.

### Data acquisition

Scanning was performed on a 7 T Siemens Terra system using a 1Tx/24Rx cervical spine coil (MRI.TOOLS GmbH). The position of the vertebrae inside the phantom was visually examined with a localizer at the beginning of the acquisition, and the vertebrae were repositioned to resemble their orientation in subject 4. At the start of each session the transmit coil reference voltage was manually adjusted both in the phantom and in vivo as previously described [9, 31]. In brief, a turbo-FLASH B_1_^+^ map was acquired and the actual flip angle within an ROI inside the spinal cord at vertebral level C4 was measured. The transmit reference voltage was then set to match the nominal and the actual FA within the ROI. B_0_ shimming was performed with the automatic shim routine of the MR system, setting the “brain” option. The shim system comprised up to full 2nd order and partial 3rd order (Z3, Z(X2–Y2), Z2X, Z2Y) shimming. The B_0_ shim region was defined to encompass vertebral levels C1 to T1, extending approximately 1 cm beyond the vertebrae in all directions, while excluding the longer spinous processes.

The acquisition protocol consisted of a high-resolution 3D MP2RAGE [9] sequence to segment the vertebrae with high accuracy, 2D B_0_ field mapping (FM) sequences to characterize static and dynamic field variations and 2D multi-echo GRE (ME-GRE) sequences to evaluate their effect on image quality. An overview of the acquisitions and their imaging parameters is given in Table 1. The MP2RAGE sequence was only acquired in vivo. A trace from the vendor-provided respiratory belt was recorded for all subjects during the acquisition.

**Table 1:** Overview of imaging parameters for the different acquisitions

	Ori	PE	Slices	Sli. (mm)	Res. (mm^2^)	FOV (mm^2^/mm^3^)	TE (ms)	TR (ms)	TI (s)	FA	R	Rep	T_acq_ (min:s)
MP2RAGE	Cor	RL	–	–	0.7 iso	260 × 171x134	2.15	5000	0.7/2.4	4/5°	2	1	8:47
Static FM	Sag	AP	70	1	1 × 1	192 × 192	4.08/5.1	ph:600iv:550–684	–	ph:42°iv:46–54°	1	2	~ 8
Breath-hold FM	Sag	AP	3	2	0.7 × 0.7	192 × 192	4.08/5.1	25	–	ph:9°iv:11°	1	1	0:14
Video FM	Sag	AP	1	3	3 × 3	192 × 156	3.45	7.3	–	ph:5°iv:6°	2	200	0:55
ME-GRE	Tra	AP	8	3	0.3 × 0.3	128 × 128	4.5/8.8/13 /17.3/21.6	264	–	ph:29°iv:35°	2	1	4:42
ME-GRE (cont.)	Tra	AP	24	3	0.3 × 0.3	128 × 128	4.5/8.8/13 /17.3/21.6	790	–	ph:48° iv:53°	2	1	3:32
Ref. GRE	Tra	AP	8	3	1 × 1	196 × 200	4.56	75	–	ph:16°iv:18°	1	1	0:17
Ref. GRE (cont.)	Tra	AP	24	3	1.2 × 1.2	226 × 230	3.59	250	–	ph:28°iv:35°	1	1	0:52

To measure the static field inhomogeneity, a sagittal-oblique 2D field map (hereafter referred to as static field map) was acquired during free breathing in vivo and without the respiratory system in the phantom. The sequence calculated the field map from two TEs acquired in separate TRs, and 2 repetitions were obtained to increase signal-to-noise ratio (SNR). The TR and flip angle (FA) were set for time efficiency and SNR maximization respectively, while considering the specific absorption rate limitations for each subject. The slab was centered on the spinal canal at level C4.

To measure the spatial distribution of the breathing-induced B_0_ fields, two sagittal-oblique 2D field maps were acquired in vivo during breath-holds, following inhalation and exhalation. In the phantom, three staples per tube were used for this acquisition, as fluctuations with 2–3 times larger intensity have previously been measured during breath-holds as compared to free breathing [2]. The field maps were collected with the tubes kept fixed in the most caudal (inhalation) or rostral (exhalation) points of the path. The slices were centered on the spinal canal and angulated accordingly. The B_0_ shim region for this sequence was expanded symmetrically in the foot-head (FH) and anterior–posterior (AP) directions, reaching the subject’s back and vertebral level T3.

To visualize the breathing-induced field fluctuations over time, a series of fast sagittal field maps (volume TR 275 ms) was acquired during free breathing in vivo and with motion of the respiratory system in the phantom. A video was created from the acquisition, available in the Supplementary materials. Details of the data analysis to create the video can be found in Sect. 1.2 of the Supplementary materials.

To investigate the effect of the breathing-induced field fluctuations on image quality, an axial-oblique 2D ME-GRE was acquired during free breathing in vivo. In the phantom, the acquisition was obtained with and without motion of the respiratory system. Eight slices were individually positioned mid-vertebrae (C1–T1) to minimize the effect of the static fields and were angulated perpendicularly to the spinal canal to reduce partial volume effects. A navigator echo (phase stabilization) was acquired, reading out a single line through the center of k-space after the last echo in each TR, to monitor the field fluctuations during the acquisition.

To investigate signal loss due to through-slice dephasing caused by the local static field gradients, an additional axial-oblique 2D ME-GRE with 24 contiguous slices and a navigator echo was acquired during free breathing in subject 4 and without motion in the phantom. The slab was positioned perpendicularly to the spinal cord covering a rostral fraction of C2 down to the caudal half of C6.

Before each ME-GRE, a fully sampled low-resolution GRE scan was acquired with the same slice geometry, to be used as reference data in the offline reconstruction. The reference GRE was acquired during free breathing in vivo and without motion in the phantom.

### ME-GRE image reconstruction

ME-GRE images were reconstructed offline with a conjugate-gradient SENSE algorithm [32] (MRIReco.jl implementation [33], 10 iterations, L2 regularization). Coil sensitivity maps were calculated from the GRE reference scans using an ESPiRIT algorithm [33, 34], and were masked using MRINavigator.jl. To visualize the effects of the B_0_ field fluctuations, all ME-GRE images were reconstructed with and without an offline navigator-based correction tailored for the spinal cord using MRINavigator.jl [17] (v0.1.1, Julia v 1.10.2). The ‘FFT unwrap’ approach was used in subject 3 and a manual phase unwrapping was performed in subject 2 and in the phantom.

### Data analyses

#### Local static field simulations

Cortical bone is approximately 1.4–2.4 ppm more diamagnetic than soft tissue [3, 5–7], presenting the largest reported susceptibility difference among biological tissues in the human thorax [8]. We therefore assumed the vertebrae to be the main contributors to local static fields in the spinal canal. Our approach of mimicking the local static fields with 3D-printed vertebrae further relied on a second assumption: that the susceptibility is homogeneous within the vertebrae. To evaluate these assumptions, the static B_0_ field produced by the vertebrae was simulated and compared to the acquired static field maps in vivo and in the phantom. To obtain susceptibility distributions ($\chi \left(\overrightarrow{r}\right)$) to run the field simulations, the C1–T1 vertebrae were manually segmented with ITK-SNAP [35] on the second inversion time of the MP2RAGE acquisition for each subject, and on the static field map magnitude image (1 mm^3^ isotropic resolution) for the phantom. Arbitrary susceptibility values of − 1 ppm and 0 ppm were respectively assigned to all voxels within the vertebrae and soft tissue, thereby assuming a homogeneous susceptibility distribution within the two tissue types. The field simulations were performed with a Fourier-based method [36–38], where each voxel is treated as an independent dipole, assuming that $|\chi \left(\overrightarrow{r}\right)|\ll 1$. This allows to compute the field perturbation in the main field direction (*z*-direction) as a convolution between the susceptibility distribution, and a dipole kernel, corresponding to a multiplication in the Fourier domain.

#### Comparison of simulated and measured local static fields

To quantitatively compare the simulations with the acquired static field maps, the susceptibility difference between vertebrae and soft tissue was estimated based on a linear regression between the measured and the simulated field within an ROI. Because the segmented vertebrae were derived from the MP2RAGE images for the in vivo data, a registration step was first needed to align the simulations with the acquired static field maps to account for small motion in between the acquisitions. The segmented vertebrae were registered on the static field map magnitude image for each subject using manual rigid registration (ITK-SNAP [35]) paying particular attention to vertebrae C3–C6. The resulting transformation was then applied to the simulated field maps using the resample function from SimpleITK [39]. The field simulation was performed before the registration step, in order to preserve the full resolution of the segmented vertebrae. This approach is valid as long as the transformation largely consists of a translation. However, in the presence of a larger angulation component, it would be more accurate to transform and resample the vertebral segmentation before the field simulation. The ROI for the linear regression was manually drawn on the segmented vertebrae, to cover the spinal canal from the caudal half of C3 to the rostral half of C6. This ROI was chosen due to its ease of alignment during registration, the minimal influence of residual background fields, and to exclude edge effects on the uppermost and lowermost vertebrae. Two-pixel erosion was then applied to the mask (Fig. S4). The acquired static field maps were separated into background field and local field, to remove the influence of the former on the linear regression. The background field was computed by applying a 3D gaussian filter (*σ* = 8.5 mm). The local fields were obtained by subtracting the background field, and subsequently smoothing (gaussian filter, *σ* = 0.8 mm) to reduce noise. A linear regression was then performed between the local field and the simulated field within the ROI. The simulated field maps were rescaled based on the estimated susceptibility values from the regression for each subject.

Finally, local field gradients in the *z*-direction (*z*-gradients) were computed for both the simulated and measured local field maps by computing the difference between each pair of neighboring voxels in the *z*-direction and dividing by the voxel size.

#### B_0_ fields profiles across vertebral levels

The static background field profile and the breathing-induced field fluctuations were quantified along the spinal canal, using the static field maps and the breath-hold field maps, respectively. To accommodate the different resolution and coverage of the different field maps, a mask defining the ROI for the quantification was generated separately for each. For the background field, a cylindrical ROI (10 mm diameter) centered on the spinal cord centerline [2, 40] was defined on the static field map magnitude image for each subject. Each background field map was first normalized to the mean value within the ROI, before quantification. For the breathing-induced fields, a similar ROI with a rectangular cross-section (AP x LR: 4.2 × 6 mm), centered on the spinal cord centerline [2, 40], was defined on the average of the magnitude images from the inhale and exhale breath-hold field map acquisitions. The breathing-induced fields were then quantified on the difference between the inhale and exhale field maps. For both the static background field and the field fluctuations, the signal within the ROI was averaged in the axial plane yielding a one-dimensional measure of the field offset. To examine results across subjects, a shared foot-head axis was constructed by defining the top of the C1 and bottom of the C7 vertebrae as reference points and linearly scaling each individual *z*-axis accordingly.

## Results

### Susceptibility estimates

The segmented vertebrae registered to the static field map acquisitions exhibited good visual alignment within the region of interest for the susceptibility measurement (C3–C6) in all subjects (Fig. S4). The alignment was less precise around C1–C2 and C7–T1, due to inter-scan motion, especially in subjects 2 and 4.

An average susceptibility difference between the vertebrae and surrounding tissue of − 1.3 ± 0.1 ppm (range: − 1.23 ppm to − 1.44 ppm) was estimated in vivo, whereas the phantom estimate yielded a lower value: − 0.41 ± 0.01 ppm (Fig. S4). All linear regressions between measured and simulated field maps exhibited a highly significant correlation (*p* < 0.001), indicating that the vertebrae are major contributors to the local static field inhomogeneity inside the spinal canal. The *R*^2^ values ranged from 0.35 to 0.62, corresponding to a moderate level of variability in the data. The regression residuals appeared mostly normally distributed, suggesting that the variability is in large part attributable to random noise (Fig. S5).

### Static field inhomogeneity

**Fig. 2:**
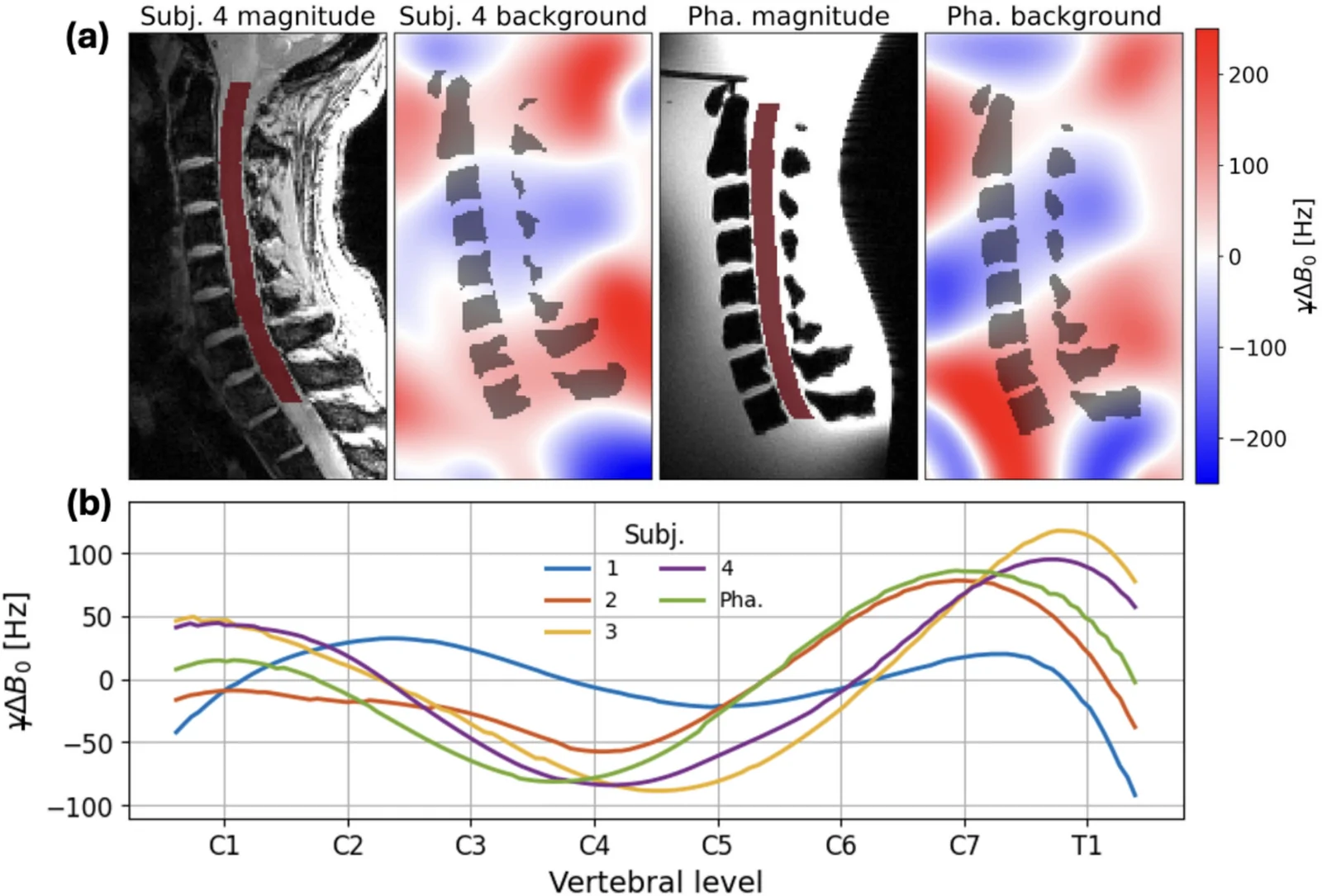
The static background fields are shown in (**a**) for subject 4 and the phantom. The magnitude images are displayed as anatomical references, and the vertebrae segmentations are overlaid on the field maps for the same purpose. A region of interest (ROI), centered on the spinal cord centerline, is overlaid (in red) on the magnitude image. The background field profile within the ROI across vertebral levels is shown in (**b**)

The background field within the spinal canal showed a consistent pattern across all subjects, with a positive field shift in the upper and lower cervical spine and a negative field shift in between (Fig. 2a). The phantom exhibited a similar background field in the spinal canal, as shown by the field profiles across vertebral levels (Fig. 2b).

**Fig. 3:**
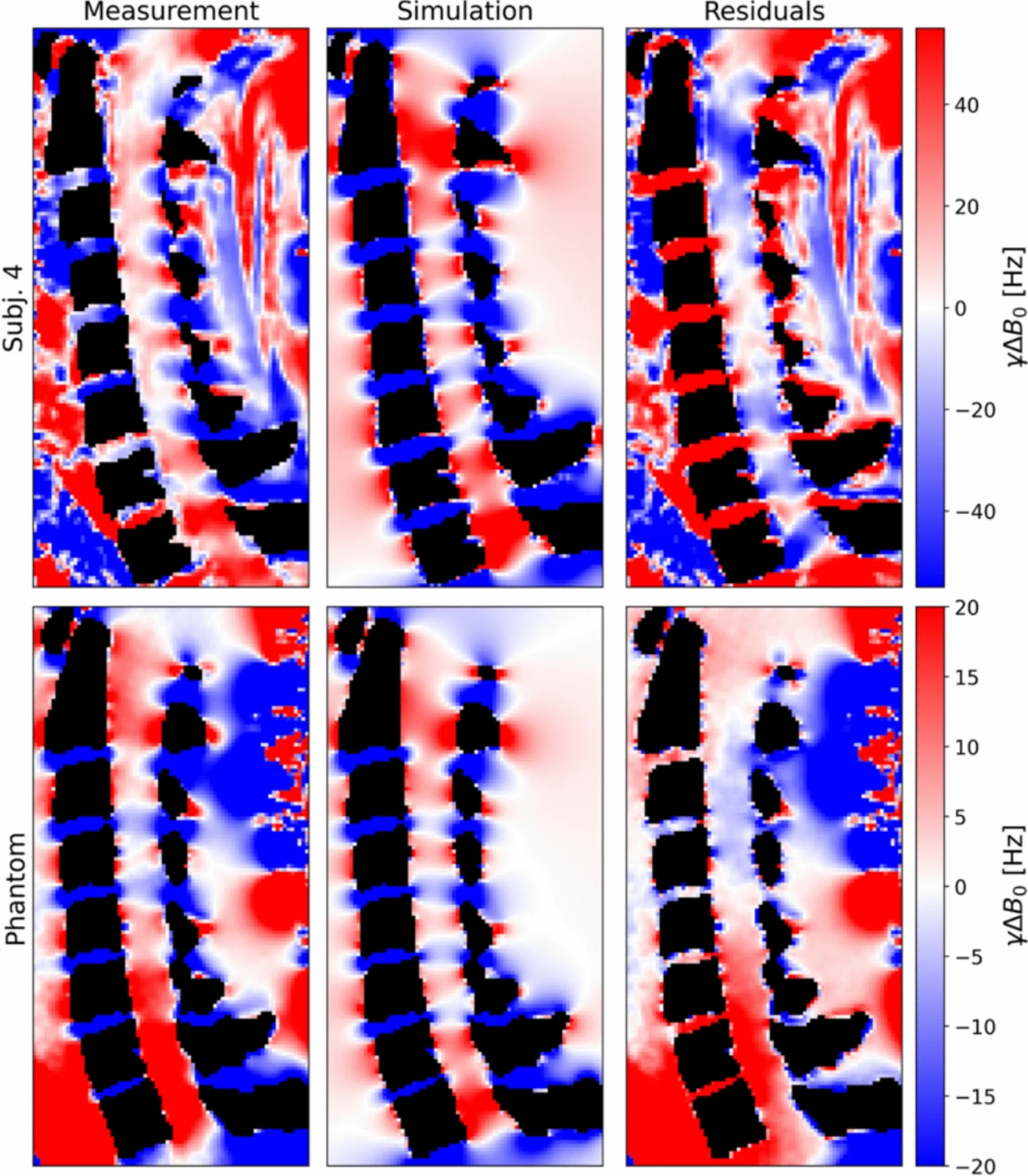
Static field maps after background field removal in subject 4 and the phantom, displayed together with the field simulations and their difference (residuals). Different scales were used for the in vivo and phantom images due to the lower susceptibility difference between the phantom vertebrae and the solution. Figure S6 shows similar results for the other subjects

The static local field pattern in the spinal canal showed similar systematic features in all subjects (Figs. 3, S6). The field was shifted negatively in the intervertebral spaces, while positive field shifts were observed on the sides of the vertebrae, creating an alternating field pattern, most strongly observed along the dorsal rim of the spinal canal. The intensity of the pattern varied across subjects and vertebral levels. Simulations based on the vertebral segmentations produced qualitatively similar patterns of alternating fields. Maps of the residuals suggest that the simulations somewhat overestimate the field variations along the ventral rim of the spinal canal in all subjects, while slightly underestimating the field variations along the dorsal rim in some subjects. The local field pattern observed in vivo was successfully replicated in the phantom, however, with approximately one-third of the intensity due to the lower susceptibility difference. The measured and simulated fields agreed very well in the phantom, apart from a residual background field component. Note that residual background fields, appearing as a smoothly varying field offset along the spinal canal, are more visible in the phantom data than in vivo due to a difference in scaling of the colormap.

**Fig. 4:**
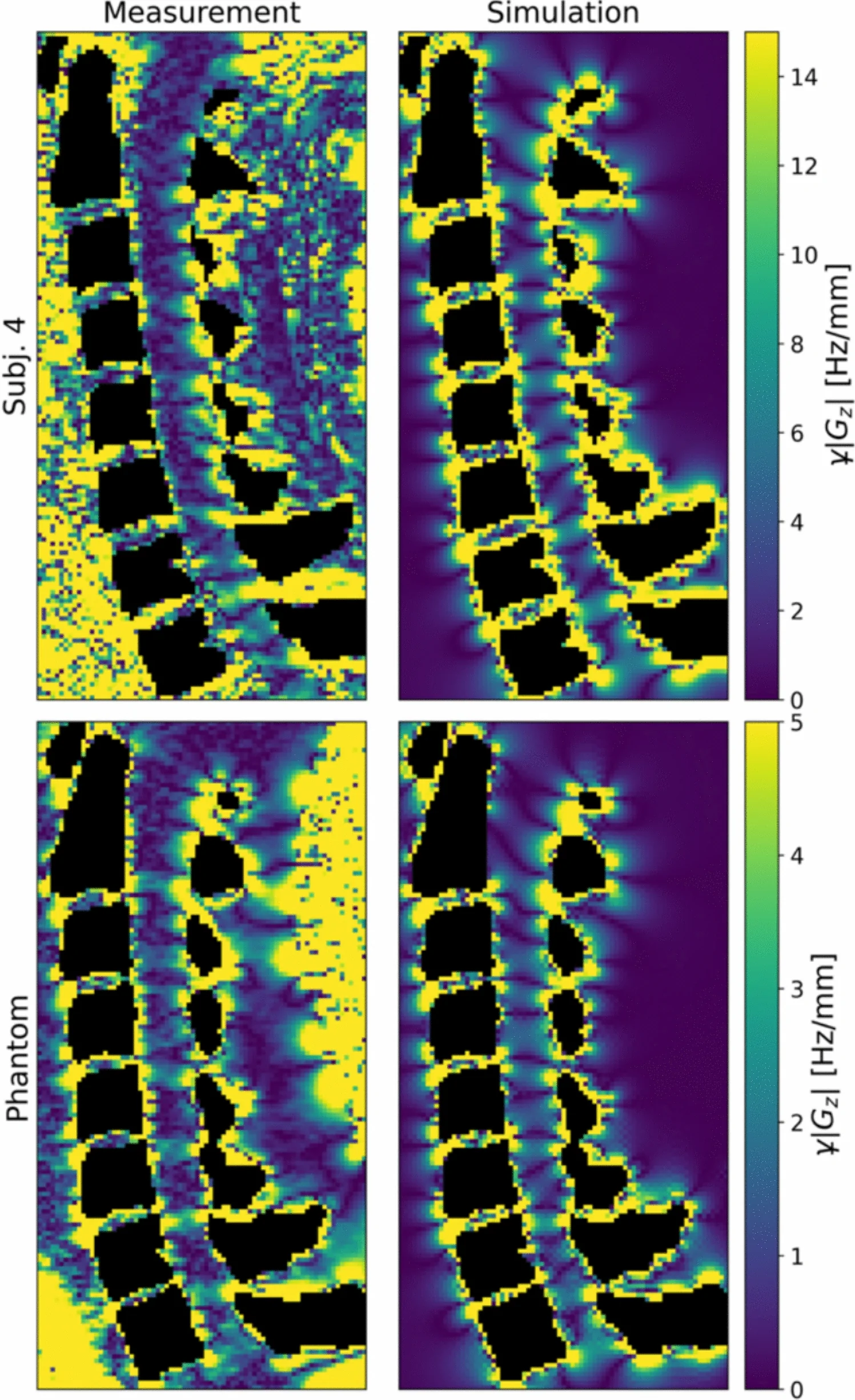
Maps of local *z*-gradients derived from the static field maps after background field removal and simulations in subject 4 and in the phantom. Different scales were used for the in vivo and phantom images due to the lower susceptibility difference between the phantom vertebrae and solution. Figure S7 shows similar results for the other subjects

The absolute value of the *z*-gradient also showed an alternating pattern along the spinal canal, with higher intensities near intervertebral spaces, especially in the dorsal part of the spinal canal, and lower intensities close to the vertebral body mid-vertebrae (Figs. 4, S7). The largest *z*-gradients were observed dorsally just above and just below the intervertebral spaces. This pattern was consistent across all subjects and the phantom. Moreover, the *z*-gradient maps showed approximately flat areas with near-zero intensity, corresponding to regions of peak field intensity, oriented almost perpendicularly to the spinal canal. These areas are easiest to identify in the simulations.

**Fig. 5:**
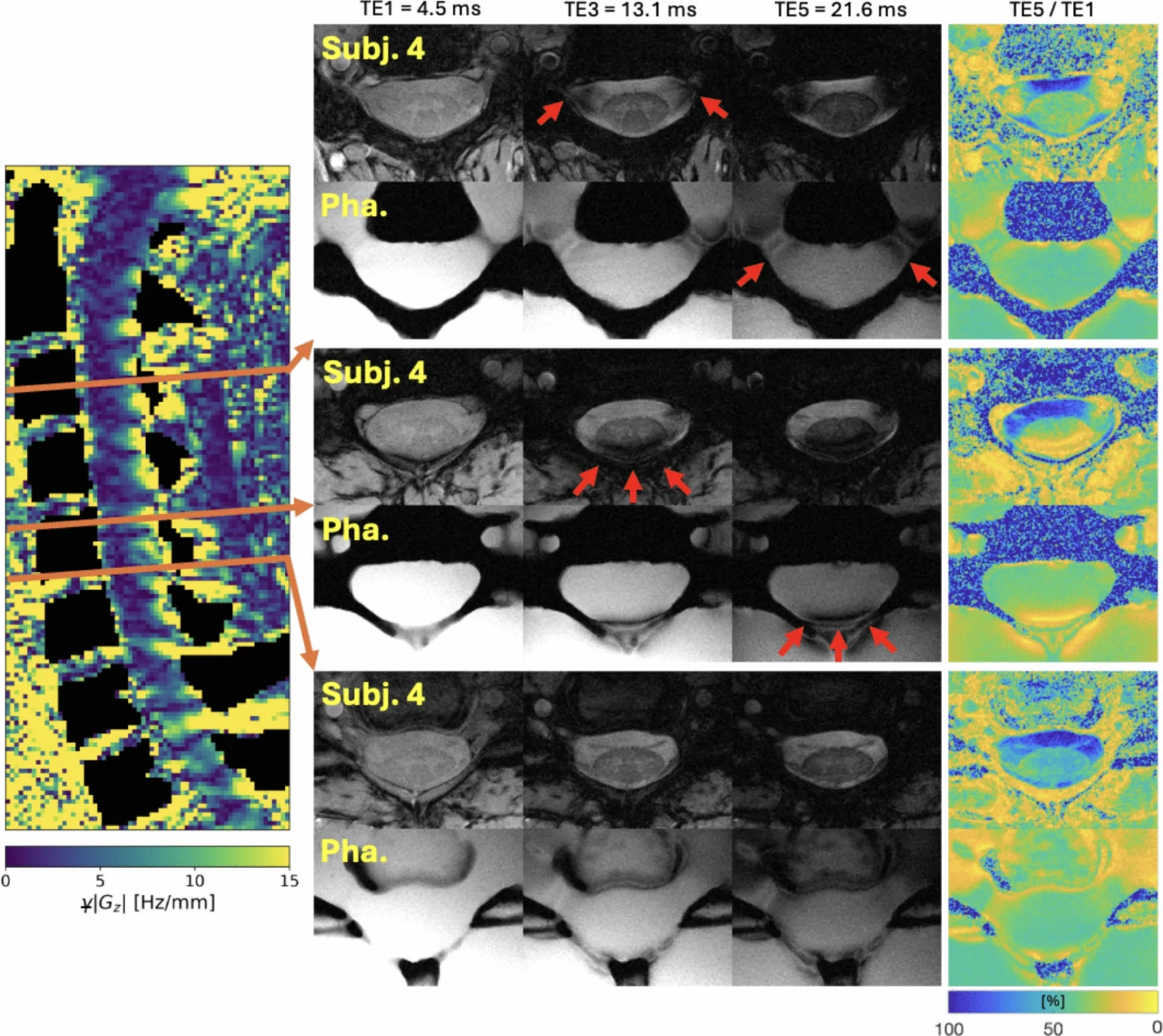
Multi-echo GRE images displaying common spatial patterns of signal dropout due to static field inhomogeneity both in subject 4 and in the phantom (Pha.). Three echoes are shown (TE1 = 4.5 ms, TE3 = 13.1 ms and TE5 = 21.6 ms) across three axial slices. The top slice, located at mid-C3, shows signal dropouts on the sides anteriorly (red arrows). The middle slice, located at the top of C5, exhibits signal dropout along the posterior rim of the spinal canal (red arrows). The bottom slice, located in the lowermost part of C5, shows homogeneous signal within the spinal canal, with negligible dropout even at later echoes. Arrows high-lighting areas of signal loss are inserted at different TE in vivo and in the phantom to indicate images with comparable degree of signal loss. To better visualize the dropout patterns, the difference between the first and last echoes was calculated and was normalized by the first echo

Figure 5 shows multiple echoes of three representative ME-GRE slices depicting the most common trends of signal loss (subject 4 and phantom) due to static local field gradients. The in vivo data were navigator-corrected to minimize ghosting from breathing-induced field fluctuations. The signal loss increased at later TE, as expected. The spatial distribution of the signal loss was more evident in the difference image between the first and last echo. Axial slices of the *z*-gradient maps at approximately corresponding slice locations visually correlated with the signal loss. The periodic nature of the local *z*-gradient yielded similar and recognizable patterns of signal loss at corresponding positions across different vertebral levels both in vivo and in the phantom. A slice positioned mid-vertebra displayed signal loss appearing anteriorly on the sides. In another slice positioned just below the intervertebral junction, the signal loss was more intense and followed the posterior rim of the spinal canal. By contrast, in a slice positioned along one of the areas with near-zero *z*-gradients, in this case right through one intervertebral junction, minimal signal dropout was visible even at later echoes. The spatial patterns were successfully reproduced in the phantom, although the lower intensity of the local gradients compared to in vivo yielded less intense signal loss.

### Breathing-induced field fluctuations

**Fig. 6:**
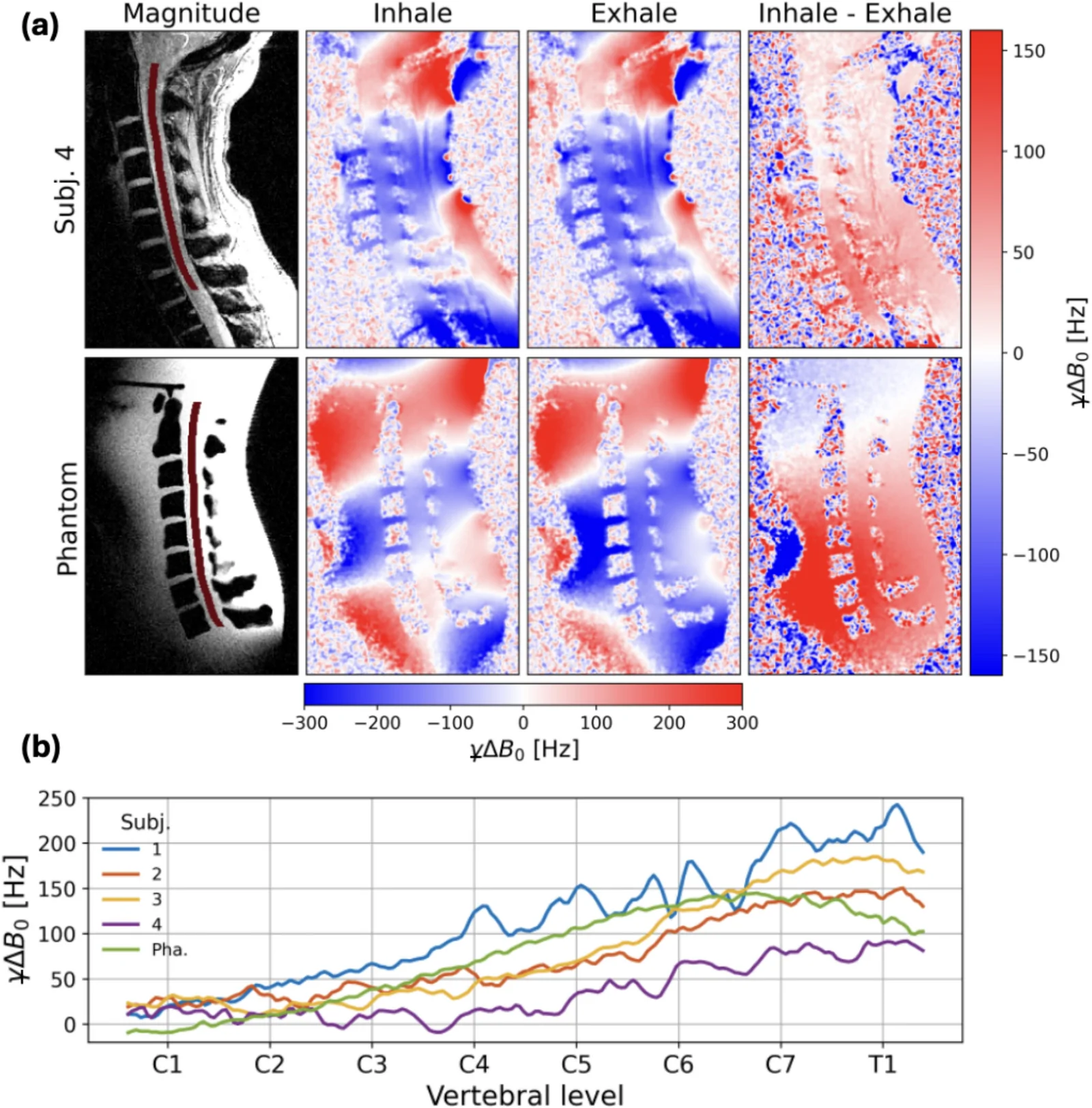
B_0_ field maps acquired during breath-holds after inhalation, after exhalation, and their difference are shown in (**a**) for subject 4 and the phantom. The second echo of the exhale field map magnitude is shown as an anatomical reference. The profile of the breathing-induced B_0_ field fluctuations (inhale–exhale) across vertebral levels for all subjects and the phantom is shown in (**b**), computed within a region of interest centered on the spinal cord centerline (in red on the magnitude image in (**a**))

Figure 6 shows field maps acquired during breath-holds after inhalation and exhalation in vivo, along with a difference image highlighting the changes between the two breathing states. The difference appeared as a smooth, spatially varying field offset. A similar field offset was replicated in the phantom using the respiratory system. The spatial profile of the field offset measured along the spinal cord centerline was on average 20 Hz (range: 14–24 Hz) around C1 in vivo, steadily increasing towards lower vertebral levels, reaching on average 154 Hz (range: 83–212 Hz) at the peak around T1. In the phantom, the field offset around C1 was about −5 Hz reaching a peak of 140 Hz at C6–C7 and decreasing to 118 Hz around T1.

**Fig. 7:**
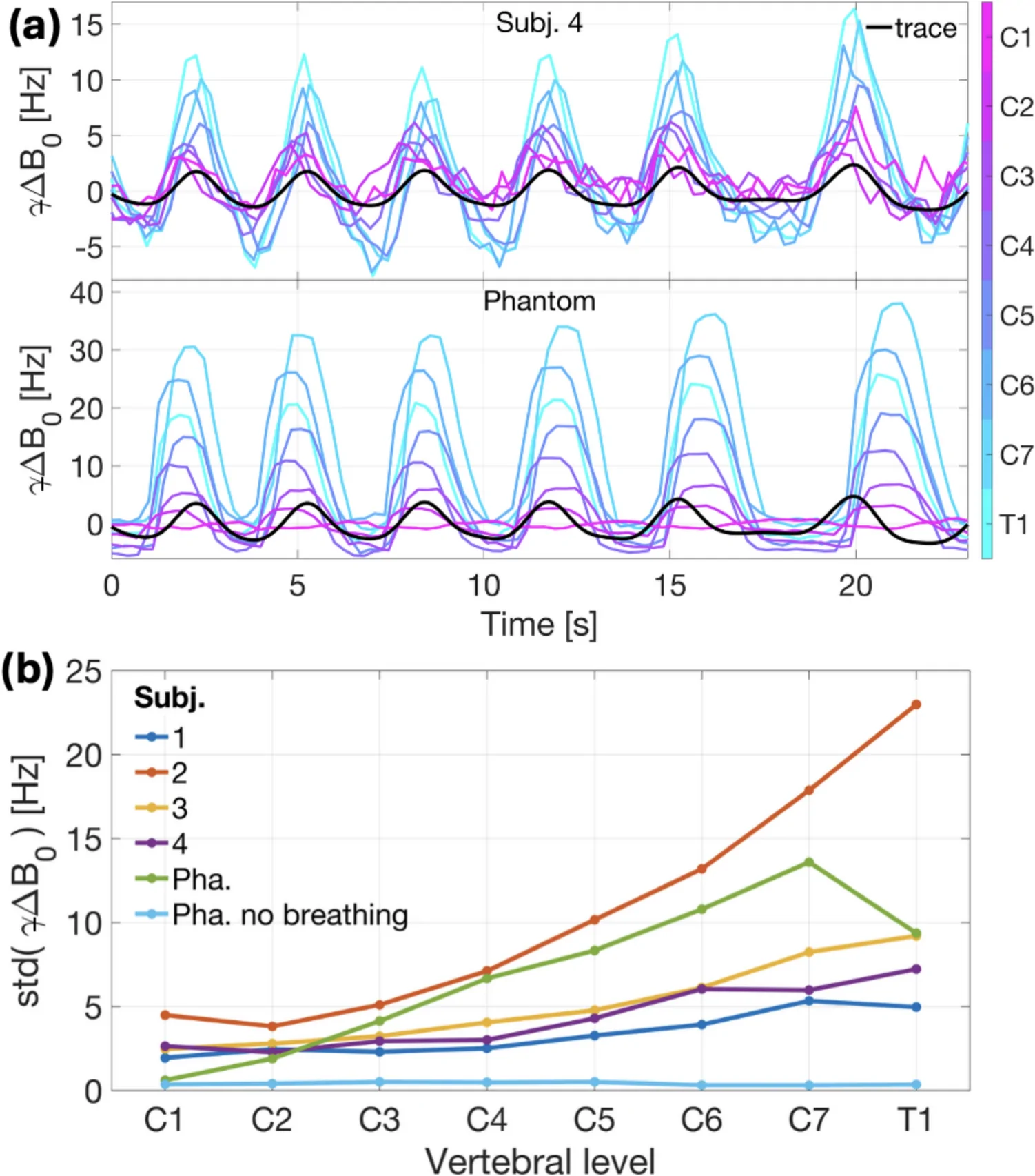
Estimates of the breathing-induced B_0_ field fluctuations measured during free breathing obtained from the phase of the navigator readout of the ME-GREs. The navigator estimates over time for all examined vertebral levels (C1–T1) in subject 4 and the phantom are shown in (**a**). The respiratory belt trace is overlaid in black. The profile of the intensity of the fluctuations across vertebral levels, quantified as the standard deviation of the navigator estimates over time for all subjects and the phantom (Pha.), is shown in (**b**). An additional profile called “Pha. no breathing” is shown, corresponding to a phantom acquisition during which the respiratory system was not moving

The phantom respiratory system was also able to approximately replicate the time course of the breathing-induced field fluctuations observed in a subject. A video showing the fluctuations in subject 4 and the phantom is available in the supplementary materials. The navigator field estimates acquired during the ME-GRE acquisition (Fig. 7a) showed similar results. The breathing-induced fields measured in subject 4 were successfully replicated in the phantom with approximately the same intensity variations and periodicity, but with rounder peaks. The respiratory system produced field fluctuations of similar characteristics during the full course of the ME-GRE acquisitions. However, phase shifts due to the motor and circuit limitations accumulated over time, resulting in a misalignment between the phantom and the respiratory system’s breathing phases after about 20 s. The magnitude of the field fluctuations, measured as the standard deviation of the navigator estimates over time, followed a similar profile across vertebral levels as for the breath-holds (Fig. 7b). In vivo the magnitude around C1 was 2.9 Hz (range: 1.9–4.5), steadily increasing to 11 Hz (range: 5–23) at the peak around T1. In the phantom, 0.6 Hz was measured around C1 and 14 Hz at the peak around C7 (9 Hz around T1).

**Fig. 8:**
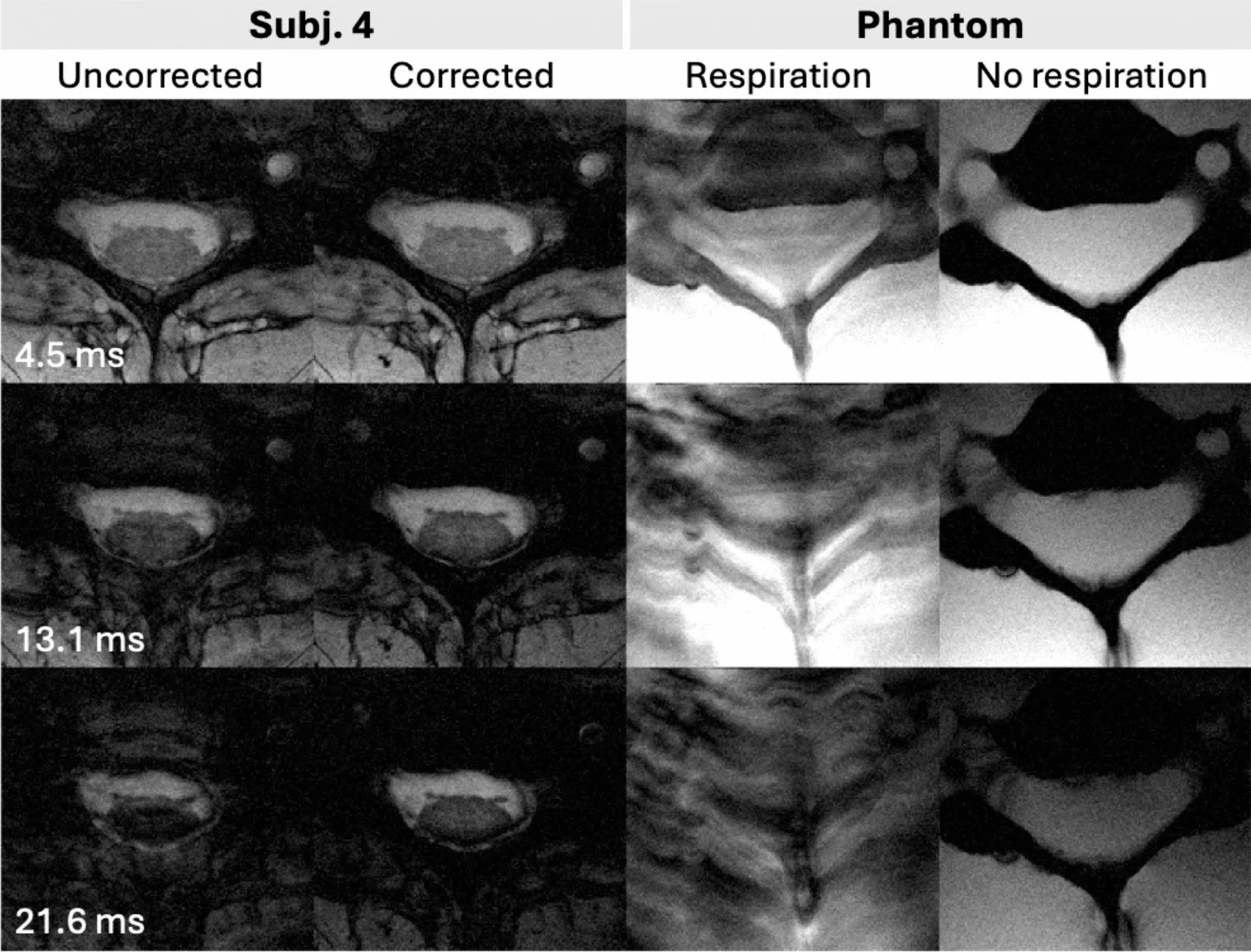
Multi-echo GRE images acquired in vivo (subj. 4) and in the phantom, illustrating the presence and absence of breathing-induced artifacts. In vivo images were acquired during free breathing and reconstructed with and without navigator-based correction. In the phantom, two acquisitions were performed: one with the respiratory system in motion and one while it was stationary. Three echoes of a single slice positioned at mid-C6 are shown for subj. 4 and the phantom

**Fig. 9:**
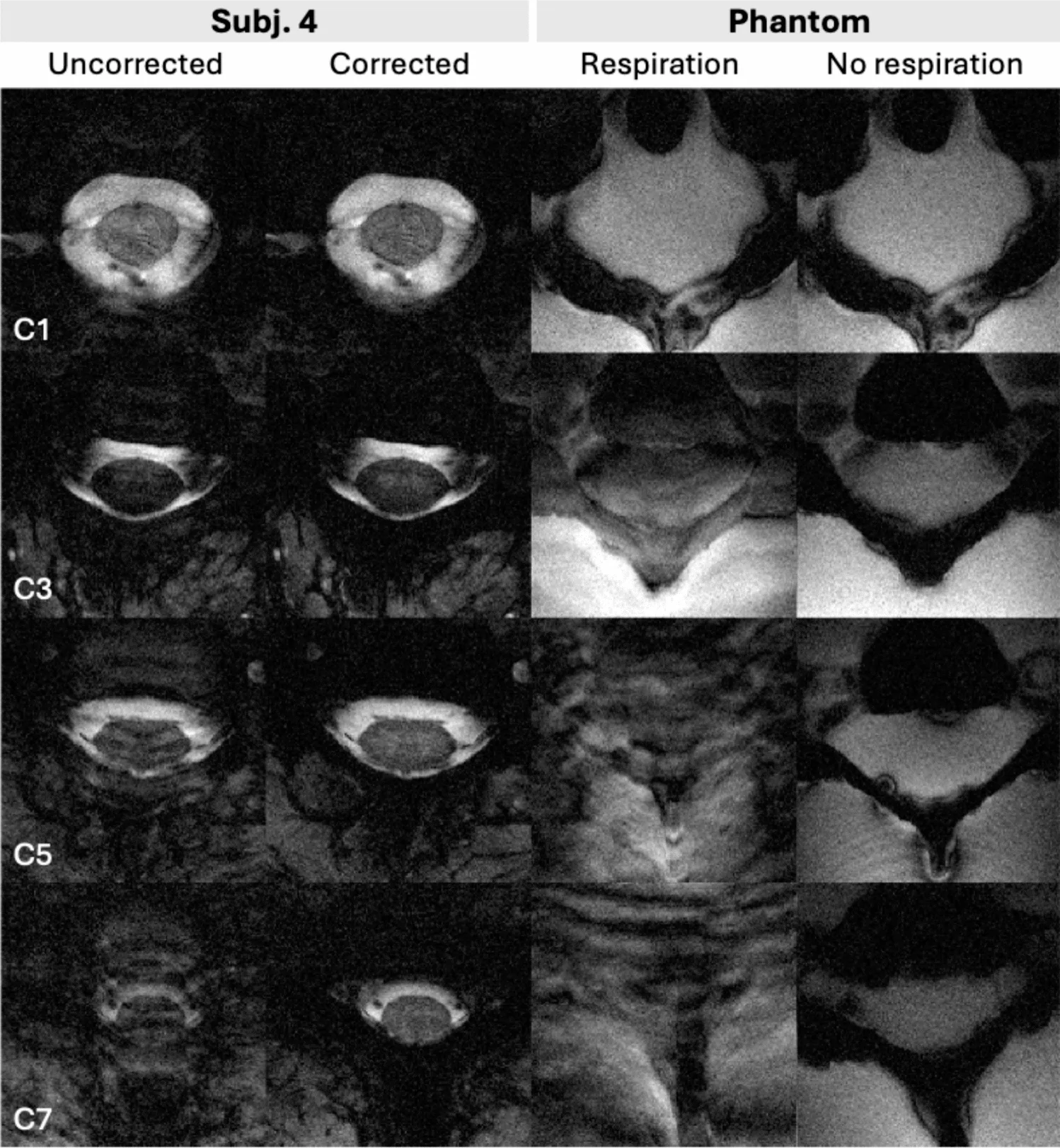
Multi-echo GRE images acquired in vivo (subj. 4) and in the phantom, illustrating the presence and absence of breathing-induced artifacts. In vivo images were acquired during free breathing and reconstructed with and without navigator-based correction. In the phantom, two acquisitions were performed: one with the respiratory system in motion and one while it was stationary. The last echo (TE = 21.6 ms) of multiple slices positioned at mid-vertebral levels (C1, C3, C5, C7) is shown for subj. 4 and the phantom

Figures 8 and 9 show multiple slices and echoes of ME-GRE images acquired during free breathing in subject 4 and in the phantom. The breathing-induced field fluctuations caused incoherent ghosting in the images with similar features both in vivo and in the phantom. The ghosting artifacts led to decreased signal uniformity within tissues, reduced contrast between tissues, and increased overall appearance of noise. Images acquired with longer TEs (Fig. 8) and vertebral levels with larger field fluctuations (Fig. 9) visually showed a higher artifact load.

## Discussion

In this study, we developed an anthropomorphic phantom with the aim of mimicking both the static and dynamic B_0_ field variations encountered in the cervical spinal cord in vivo, and their effect on T_2_*-weighted GRE acquisitions. Such a phantom could aid the development of advanced acquisition and correction techniques to tackle the persistent challenge of B_0_ field variations in the spinal cord. The phantom consisted of a 3D-printed shell in the shape of a human head, neck and thorax, allowing replication of the static background B_0_ field distribution with high fidelity. The phantom shell can easily fit in a commercial cervical spine coil, enabling scanning at 7 T. The shell was filled with a uniform liquid solution, into which 3D-printed vertebrae were inserted to approximate the spatial distribution of the local static B_0_ field inhomogeneity but with lower magnitude than in vivo. Finally, an external vest was constructed through which iron staples were moved to create temporal B_0_ field fluctuations, which allowed replication of key characteristics of breathing-induced field fluctuations in vivo. The phantom design was additionally driven by a number of key features, which served to guide design choices.

First, the phantom was designed to be low cost. The total cost of the phantom was around €1700 (excluding costs related to the respiration vest and motor support components which were sourced in-house). While most of the design and construction were performed in-house, the vertebrae and phantom shell were printed by professionals due to the need for specific materials and technical expertise. They also handled the assembly of the phantom shell’s jigsaw puzzle, ensuring precise alignment and structural solidity. To reduce the cost of 3D-printing, the shell was resized to 80% in each dimension. The resizing also reduced its weight (from ~ 20 L to ~ 10 L volume), making the phantom easier to handle practically at the scanner. The resized phantom had a neck circumference of approximately 31 cm, which is around the median for a slim female adult, whereas the original model had a neck circumference of ~ 38 cm, corresponding to a large adult male [41]. The size of the phantom shell may affect the profile of the background B_0_ field relative to the vertebrae, and may also influence the B_1_ distribution. The 3D-printed vertebrae were not resized, but originated from a rather small subject, such that the size relationship between the shell and the vertebrae was not disproportionate.

Furthermore, the phantom was designed to be modular and reproducible, offering a high degree of adaptability. To enable intra-session adjustments to the phantom, e.g. repositioning or replacing the vertebrae, it was essential to make it free from biological or chemical hazards. Hence, we decided on a non-toxic liquid solution, deliberately avoiding any gelling agents or toxic additives. Consequently, the solution can be easily changed, providing the opportunity to tune its properties to meet specific needs. However, the requirement of a non-toxic liquid solution also limits the range of additives available for tuning. We made use of salt to tune conductivity, and sugar to tune permittivity and T_2_* relaxivity. Because we focused on ME-GRE acquisitions, it was important that the T_2_* was approximately in the range of human tissues. Increasing sugar reduces T_2_* values, effectively setting an upper limit to the sugar concentration. With the sugar concentration used for the phantom, the T_2_* was ~ 22 ms and the relative permittivity is estimated at around 74 [42]. This permittivity is higher than for average human tissues in the region [43], which may affect the B_1_ distribution in the phantom, making it less comparable to the in vivo case. The conductivity is estimated at 0.64 S/m [42], which is realistic for human tissue.

Moreover, we found in preliminary work [44] that the sugar shifted the susceptibility of the phantom solution towards more diamagnetic values, which reduced the susceptibility difference between the solution and the 3D-printed vertebrae. The estimated susceptibility difference between the vertebrae and the phantom solution was approximately one-third of the values estimated in vivo. One potential way to increase this difference is by dissolving paramagnetic salts, such as manganese chloride, into the solution [45]. However, this would alter the solution’s permittivity, conductivity and T_2_ [46]. Furthermore, these salts are hazardous and must be handled under a fume hood, which limits the feasibility of intra-session modifications to the phantom. Future optimization of the phantom may include investigation of different non-toxic additives to simultaneously match conductivity, permittivity, relaxivity and susceptibility to realistic values for human tissues. MR phantom fillings commonly also include gelling agents [29, 47] to reduce diffusivity as well as T_1_ and T_2_ relaxation times. A phantom filling recipe that includes a gel may be preferable when matching relaxivity and diffusivity is prioritized over phantom flexibility [18, 29].

Finally, soap was added to avoid air bubbles attaching to the surface of the 3D-printed vertebrae, which strongly impacted field maps. While this served our purpose and aligned with the flexibility we required, the air bubbles may be more consistently prevented by adding a degassing procedure after phantom filling.

The approach of 3D-printing vertebrae to replicate the local static B_0_ field inhomogeneity, relied on two main assumptions: that the vertebrae are the main cause of the local static fields, and that the magnetic susceptibility of the vertebrae is homogeneous. The close resemblance between the acquired field maps and the field simulations validates the first assumption. However, the maps of the residuals also give a hint as to where the model is insufficient, particularly in regard to the second assumption. In reality, the vertebrae are not of homogeneous bone tissue. While the thin arcs and processes largely consist of cortical bone, the vertebral bodies are mostly made up of trabecular bone. Trabecular bone is less diamagnetic [4, 48] than cortical bone and contains bone marrow rich in fat [5], which is more paramagnetic than other soft tissues. Studies of cortical bone in different body parts have arrived at values in the range of −1.4 ppm to −2.4 ppm relative to soft tissues [3, 5–7], whereas studies of vertebral bodies yielded a range of values closer to soft tissue, depending on trabecularization and fat content [49]. Incorporating these variations in tissue composition of the vertebrae in the simulations may enhance the model’s accuracy. Similarly, intervertebral discs, nerves, spinal cord, and other soft tissues could be added to the model, as soft tissues also exhibit varying susceptibility values [8]. The simulated field has a range of potential applications. While we used it to evaluate our assumptions and estimate susceptibility differences, it could also be applied in the development of advanced shimming techniques for spinal cord imaging.

Given the variability in bone tissue within the vertebrae, it was not a priori clear which susceptibility difference to target for the phantom. Based on the simulations, we estimated the susceptibility difference between the vertebrae and surrounding soft tissues to − 1.3 ± 0.1 ppm in vivo, which is compatible with the value measured in cortical bone of the forearm by Dimov et al., [3] using quantitative susceptibility mapping (− 1.4 ± 0.2 ppm). However, we observed that the fitting results were highly sensitive to signal processing parameters, including the number of voxels eroded from the spinal canal mask, the mask’s placement, and the standard deviation of the gaussian filters used to remove background fields and reduce noise. Therefore, the processing parameters were individually tuned to ensure that small variations in their value did not significantly affect the results. The width of the gaussian filters was set conservatively to avoid altering the intensity of the local static fields generated by the vertebrae, thereby preserving the accuracy of the susceptibility estimation. Alternatively, other background removal techniques, developed for quantitative susceptibility mapping, could be used [50]. In the end, the estimated susceptibility values primarily served as an approximate guide for the phantom construction.

3D printing has garnered growing interest from the scientific and clinical communities for constructing medical phantoms [20, 23, 24, 28, 51–53], due to its versatility and affordability. While most of the previous work did not consider the magnetic susceptibility of the 3D-printed material, investigations have been undertaken to chart the magnetic susceptibility of various printing materials [28], to minimize unwanted static B_0_ field inhomogeneity. Even with the most diamagnetic material from that study, we still did not reach the targeted susceptibility difference. Yet several alternative printing materials and techniques exist and continue to be developed that could be explored further. Additionally, it is possible to further customize the printing materials with additives [54, 55]. For our purpose, it may for example amount to adding calcium salts to make the 3D-printing resin more diamagnetic. In an extended model, materials with different features could also be used to print various tissues such as cortical bone, trabecular bone with bone marrow, and intervertebral discs. Regarding the phantom shell, adoption of an alternative printing process and material could yield a more solid, single-piece shell, eliminating the need for epoxy and fiberglass reinforcement. Indeed, even though the effect of the coating was not observed in the acquired data, the coating process was tedious and person-dependent, thereby reducing the phantom’s reproducibility.

The vertebrae were fabricated using stereolithography (SLA) 3D printing, a process that generates support structures to prevent warping during printing. After printing, the supports were removed but small residues remained on the surface. These residues had the potential to affect the fields generated by the vertebrae in the spinal canal. To mitigate this, we switched the printing orientation from horizontal to vertical, relocating the supports from the spinal canal to the intervertebral spaces. This adjustment reduced the impact of the residues, at the expense of increased printing time.

A more robust positioning system for the vertebrae could have been integrated into the phantom shell design. However, it would be challenging to design such a system that also accommodates different vertebrae sets with varying lengths, shapes and orientations. Hence, we opted for a more flexible but less rigid system based on threads. This allows vertebrae to be replaced and repositioned as needed, which could for example enable investigation into how the fields vary with different angles between the vertebrae and the main field. However, precise placement can be challenging due to limited visibility in the confined space within the phantom. Consequently, this limits the reproducibility of the positioning. Important to note is that buoyancy varies with printing material density, affecting whether the vertebrae float or submerge. Thus, the anchor points should be placed anteriorly or posteriorly within the phantom depending on the material used.

The respiration system enabled successful replication of the breathing-induced field fluctuations encountered in a specific subject in vivo, as well as the resulting ghosting artifact in ME-GRE acquisitions. Using a non-elastic thread to connect the staples to the motor was essential for ensuring the system’s effectiveness. This design choice enabled the staples to respond immediately and precisely to the motor’s movements, eliminating any delay or slack that could compromise performance. The waveguide that the threads passed through was located directly in front of the bore, which helped reduce friction. This alignment between the waveguide and the scanner bore is, however, not present at all sites. With our setup, it was possible to adjust the rate, depth and shape of the breathing. The depth of breathing was controlled via the range of motion and number of staples in the tubes. The shape of the breaths was controlled via the motion of the motor, which was given a respiratory trace from a specific subject as input. This approach relies on a linear relationship between the respiratory trace and the field fluctuations in vivo, and between the position of the staples and the field shift in the phantom. Neither is strictly true, but it is a sufficiently close approximation to generate realistic breathing waveforms in the phantom. Due to the electronics and motor characteristics of our setup, it was necessary to restrict the speed to 60 RPM, which limited the maximum slope of the field fluctuations in the phantom, and resulted in a phase shift between in vivo and phantom breathing cycles. This issue could be addressed by incorporating higher-performance electronic components. Precise synchronization with the scanner was not central to this study, and we therefore did not implement triggering of the respiratory system. However, it should be possible in principle to take a trigger input to start the breathing motion and the imaging sequence simultaneously for applications where exact control over the timing is important. Alternative implementations to move the staples may for example make use of a pneumatic system.

The dynamic B_0_ field fluctuations exhibited a consistent profile across vertebral levels compared to previously reported measurements at 3 T and 7 T [2, 10, 17]. Specifically, a prior study at 7 T reported a standard deviation of 10 Hz during free breathing and a field shift of 90 Hz between inhale/exhale breath-holds at C7 [2]. In comparison, we observed similar values during free breathing (in vivo T1, 11 Hz; phantom C7, 14 Hz) and moderately stronger field shifts between breath-holds (in vivo T1, 154 Hz; phantom C6–C7, 140 Hz). The variability between subjects is large, and differences in the study cohort could have contributed to the observed disparities. In vivo, the peak of the field fluctuation profile was positioned more caudally at T1, whereas in the phantom, it was at C6–C7. However, this difference only slightly altered the distribution of the artifact load along the spinal canal. Potentially, it could be corrected by repositioning the enclosed pathways of the phantom vest to a more caudal location. The breathing-related field fluctuations can interfere with signal spatial encoding and lead to pseudo-displacements in the images [22]. However, breathing may also cause actual displacement of the spine and the spinal cord in vivo. Differentiating between these two effects is challenging, as both result from respiratory motion. Unlike in vivo scenarios, the respiration system reproduces field fluctuations without any spinal movement, enabling a more precise evaluation of their impact.

Opportunities exist for expanding the phantom’s potential applications. While the vertebral model was derived from MR data of a specific subject to enable direct comparison of the local static field inhomogeneity, it may be replaced with a more representative model of the average healthy anatomy. Moreover, the phantom could be customized to replicate pathological conditions such as scoliosis, kyphosis, or vertebral fractures, based on the anatomy of a specific patient or patient group, enhancing its relevance for research focused on these pathologies. Vertebral segmentations can also be obtained from CT images, providing greater anatomical detail due to the higher resolution [20, 21, 23, 51]. While CT scans are less commonly performed on healthy subjects, patients are often subject to them. We focused on the cervical spinal cord (C1–T1); however, the phantom shell allows for extending the 3D-printed vertebrae to include upper thoracic vertebrae. Extending the phantom shell beyond the thorax would allow for the inclusion of additional thoracic and lumbar vertebrae, broadening its scope of use. Finally, the approach of using 3D-printed materials with different magnetic susceptibility to shape the static B_0_ field may be useful also to mimic other parts of the body.

## Conclusion

In this study, we presented a phantom mimicking the static and dynamic B_0_ field variations encountered in the cervical spinal cord in a given subject in vivo. The phantom may serve to assess novel correction techniques addressing the persistent challenge of B_0_ field variations in spinal cord imaging. The phantom’s modularity and adaptability allow for easy modifications to meet different experimental needs. While key features of the B_0_ field variations were replicated with high accuracy, the local static B_0_ fields were of lower magnitude than in vivo. Further development could enhance replication fidelity and expand the applicability of the phantom, potentially by investigating different materials for 3D-printing and incorporating additional anatomical features. Beyond spinal cord applications, the study demonstrates the potential of using 3D-printed objects to shape the local B_0_ field inhomogeneity, which may be relevant also for phantoms of other body parts.

## Supplementary Information

Below is the link to the electronic supplementary material.

Supplementary file1 (MP4 983 KB)

Supplementary file2 (PDF 4004 KB)

## Data Availability

The custom-designed 3D models are freely available on GitHub (https://github.com/NordicMRspine/Cervical_phantom_3Dmodels). The vertebrae segmentations are not included in this folder but can be provided upon request. The code developed for this study is available upon request to the corresponding author. The code for the offline navigator-based correction is available in a Julia package called MRINavigator.jl v0.1.1 (https://github.com/NordicMRspine/MRINavigator.jl, example script and expected outcomes: https://github.com/NordicMRspine/UserExample_MRINavigator/tree/main, v0.0.1). Phantom data are available upon request; in vivo data are not available due to institutional policy.
