# Supplementary material for: Towards an anthropomorphic MRI phantom mimicking static and dynamic B0 field variations in the human cervical spinal cord
Source: MAGMA. 2026 Apr 15;39(5):877–95. doi: 10.1007/s10334-026-01349-4 (PMC13719740; doi:10.1007/s10334-026-01349-4)
Supplement: Supplementary file 1 [file 10334_2026_1349_MOESM2_ESM.pdf]

Supplementary materials:
Towards an anthropomorphic MRI phantom mimicking static and dynamic B_0 field variations in the human cervical spinal cord

AUTHORS: Laura Beghini[1], Brunnhilde M. A-S. Ponsi[1,2], Kamilla Refsholt[1], Annelen Dogger Schmidt[1], Virginie Callot[3,4], S. Johanna Vannesjo[1]

1. Department of Physics, Norwegian University of Science and Technology, Trondheim, Norway
2. CRCI2NA, INSERM UMR 1307, Nantes, France and Nuclear Medicine Department, University Hospital, Nantes, France
3. Aix-Marseille Université, CNRS, CRMBM UMR 7339, Marseille, France
4. AP-HM, Hopital de la Timone, Pôle d'imagerie médicale, CEMEREM, Marseille, France

Outline:

1. Methods (page 2)
   1.1. Phantom design
   1.2. Field video acquisition and analysis
   1.3. Figures S1 - S3

2. Results (page 6)
   2.1. Figures S4 – S7

3. References (Page 10)

1. Methods

1.1 Phantom design

1.1.1 Phantom shell

The phantom shell was obtained by modifying an open-source average head and neck model [1] in Solidworks (Dassault Systèmes Solidworks Corporation, 2022). The model originally ended just below the shoulder and was extended to the chest level. To enable insertion of the vertebrae, the original side opening was replaced by a large circular opening with a screw-on-cap placed anteriorly on the thorax. A thin bar was added on the inside of the shell to serve as a positioning reference for the vertebrae. The bar was oriented perpendicular to the coronal plane and connected to the middle of the phantom's nose (see MR image of the phantom in Fig. 1). To reduce the cost of 3D-printing and the phantom weight, the model was resized to 80% of its dimensions in each direction. The shell model was divided into pieces as a jigsaw puzzle to optimize the printing process (Fig. 1) and 3D-printed in nylon (SLS printing, EOS PA2200 [2]). The printed pieces were glued together with Cyberbond 2028 [3]. To ensure that the phantom was waterproof and mechanically robust, the shell was internally coated with laminating epoxy [4], and externally reinforced with the same epoxy and roving fiberglass [5]. Once the epoxy had hardened the exterior was polished with sandpaper to obtain a smooth surface.

1.1.2 3D-printed vertebrae

The model for the vertebrae was obtained by manually segmenting C1 to T1 on a T1-weighted MP2RAGE sequence [6] (0.7 mm^3 isotropic resolution) acquired on subject 4 (male, age: 23 yo, BMI: 21.4, height: 169 cm). During the segmentation process, it was challenging to distinguish between adjoining vertebrae at the laminar surfaces and/or articular processes. As a result, the segmented vertebrae C2 to T1 were connected at these interfaces. To 3D-print the full cervical spine in one rigid piece, all vertebrae needed mechanical connections. Small support rods were therefore inserted between the C1 and C2 articular facets (Fig. S1). The model was then 3D printed (SLA printing, vertical orientation) using Rigid 10K from Formlabs [7] which is known to have negative susceptibility [8] compared to water, as expected for cortical bone compared to soft tissue [9–12]. The 3D-printed spine was positioned inside the phantom with thin cotton sewing threads passed through the transverse foramina. The threads were attached to four concrete hooks in plastic [13] that were glued to the phantom interior (two above the ears and two next to the thorax opening).

1.1.3 Phantom solution

The phantom solution consisted of water, 5 g/L of salt to match the conductivity to human tissues [14], 200 g/L of sugar to reduce T_2* (Fig. S2) and to tune the permittivity [14], and 5 mL/L of liquid hand soap [15] to reduce surface tension in order to prevent the formation of small air bubbles around the vertebrae. The solution was prepared in advance, ensuring that all the ingredients were fully dissolved. The 3D-printed vertebrae were physically moved around a bit after being inserted into the phantom solution, to detach any remaining air bubbles.

1.1.4 Respiratory system design

To mimic breathing-induced field fluctuations in the phantom, we built an automated system to move iron staples [16] over the phantom thorax in a periodic manner. The staples were

taped inside two plastic tubes that were filled with water to absorb heat, in case eddy currents were induced. The tubes were pulled back and forth along the foot–head direction over each side of the phantom thorax with polyester silk threads (1.4 mm diameter) connected to a rotating stepper motor (NEMA17-13-04SD-AMT112S). To ensure a reproducible pattern of motion, a vest was constructed featuring two distinct (left and right) enclosed pathways with removable lids, oriented along the foot–head direction. The vest was positioned on the phantom thorax in correspondence to the lungs (Figs. 1, S3). It was designed in FreeCAD (FreeCAD Project Association, v0.20.2), and 3D printed (PLA filament at 30% infill density) in six pieces that were glued together. Two plastic concrete hooks were attached to each external side of the phantom thorax and vest. Rubber bands around the hooks were used to anchor the vest to the phantom and to secure the lids over the pathways (Fig. 1). The stepper motor was controlled by an Arduino Uno Rev3 via an SN754410ne H-Bridge (Fig. 1) (bipolar stepper circuit and schematic available online: https://docs.arduino.cc/learn/electronics/stepper-motors/). A 3D printed cylinder (PLA filament) was attached to the rotating rod of the stepper motor using an all-thread screw (3 mm diameter, with hexagonal sockets compatible with a 2 mm Allen key). The cylinder had a diameter of 6.4 cm, resulting in a circumference of about 20 cm, corresponding to the pathway lengths (Fig. S3). All electronic components were positioned on a table in the equipment room and the threads were passed through a waveguide located in front of the magnet bore. The stepper motor was anchored to the table with a 3D-printed support.

1.1.4 Respiratory system control

The respiratory belt trace acquired on subject 4 was synchronized with the MR data using the PhysIO toolbox[17]. The time interval of interest for the trace recording was selected, and the motor rotation direction and speed were calculated as follows. A full rotation of the NEMA-17 stepper motor consists of 200 steps. The amplitude of the respiratory trace acquired on subject 4 was re-scaled to range from 0 to 200. Therefore, one full rotation of the stepper motor moved the staples from the most rostral to the most caudal position within the enclosed pathways respectively corresponding to inhalation and exhalation. The motor rotates clockwise as the step number increases and counter-clockwise as the step number decreases. Due to storage limitations in the Arduino, it was determined to make the motor change speeds every second step. The module of the speed v (expressed in Revolutions Per Minute (RPM)) required for each pair of steps was computed as:

$$|v(x)|[\text{RPM}] = 2\frac{60[\text{s/min}]}{x\left[\frac{\text{s}}{\text{steps}}\right]\cdot 200\ [\text{Steps/Rotation}]},$$

where x represents the time required for the respiratory belt to complete the two steps. If the trace took more than 0.6 s to progress from one step to the next, a delay was implemented instead of adjusting the speed. The delay caused the motor to remain stationary, better reflecting the subject's natural breathing pattern. Additionally, a limit of 60 RPM was set for the speed module. This constraint originated from the limitation of the stepper motor and electronic circuit.

The code used to calculate the speeds for the stepper motor and input them into the Arduino is available upon request.

1.2 Field video acquisition and analysis

A time series of fast field maps was acquired to monitor the breathing-induced field fluctuations during free breathing in subject 4 and in the phantom. The trace recorded with the vendor-provided respiratory belt during this acquisition on subject 4 was used in input to the phantom respiratory system. The acquisition parameters are given in Table 1 (in the main text). The TR for one volume was 275 ms. The slice was centered on the spinal canal at level C4 and angulated accordingly. The B_0 shim region was set as for the breath-hold acquisitions. After the acquisition, each phase image was unwrapped using the "unwrap_phase" function from scikit-image[18]. One image, representing a neutral position in the respiratory cycle between inhalation and exhalation, was used as a reference and subtracted from all other images in the same acquisition using complex division. The phase images where then rescaled to Hertz. The phantom images were manually synchronized with the images collected on subject 4. A time interval of about 20 seconds was selected within the acquisitions and a video of the phantom and subject 4 was generated using FFmpeg[19].

1.3 Figures

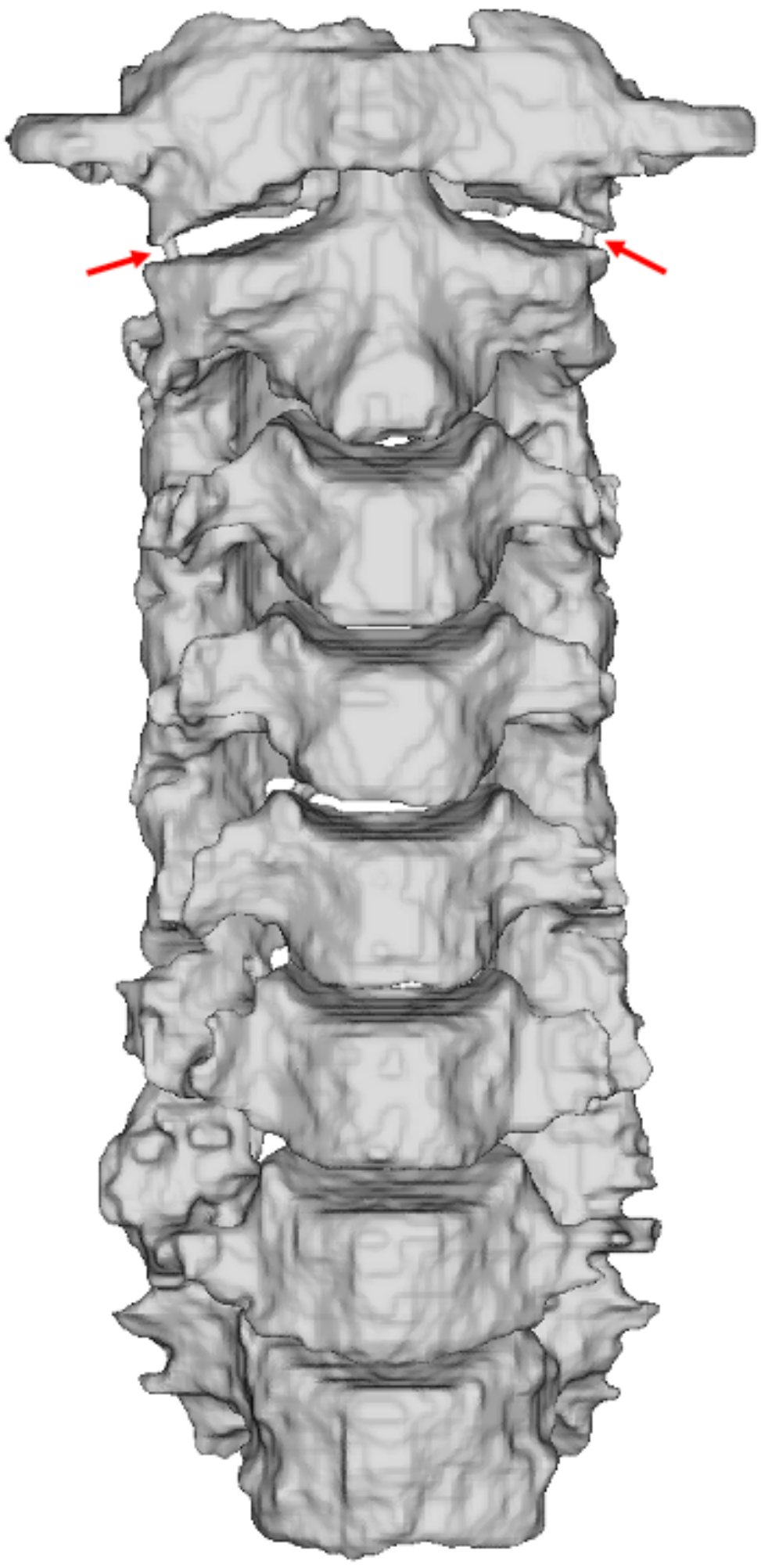

Fig. S1 Manually segmented C1 to T1 vertebrae of subject 4. Small support rods (indicated by red arrows) were inserted between the inferior articular facet of C1 and the superior articular facet of C2 to link the two vertebrae and enhance the structure's rigidity before 3D printing.

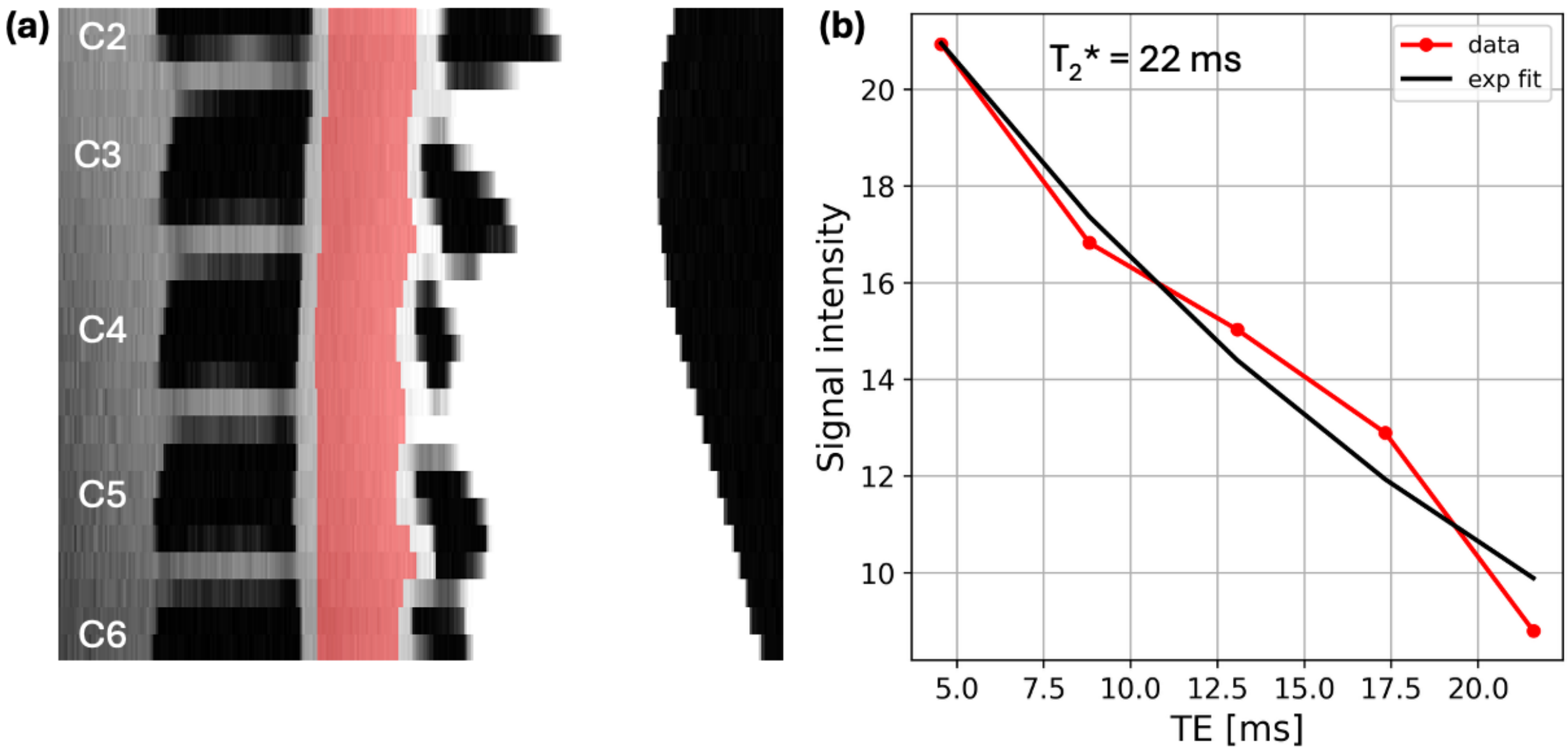

Fig. S2 T_2* estimate of the phantom solution using the multi-echo GRE sequence acquired in a single slab (5 echoes). The signal in the red region of interest (**a**) was averaged for each echo, and a mono-exponential fit (with two degrees of freedom) was applied to the data (**b**).

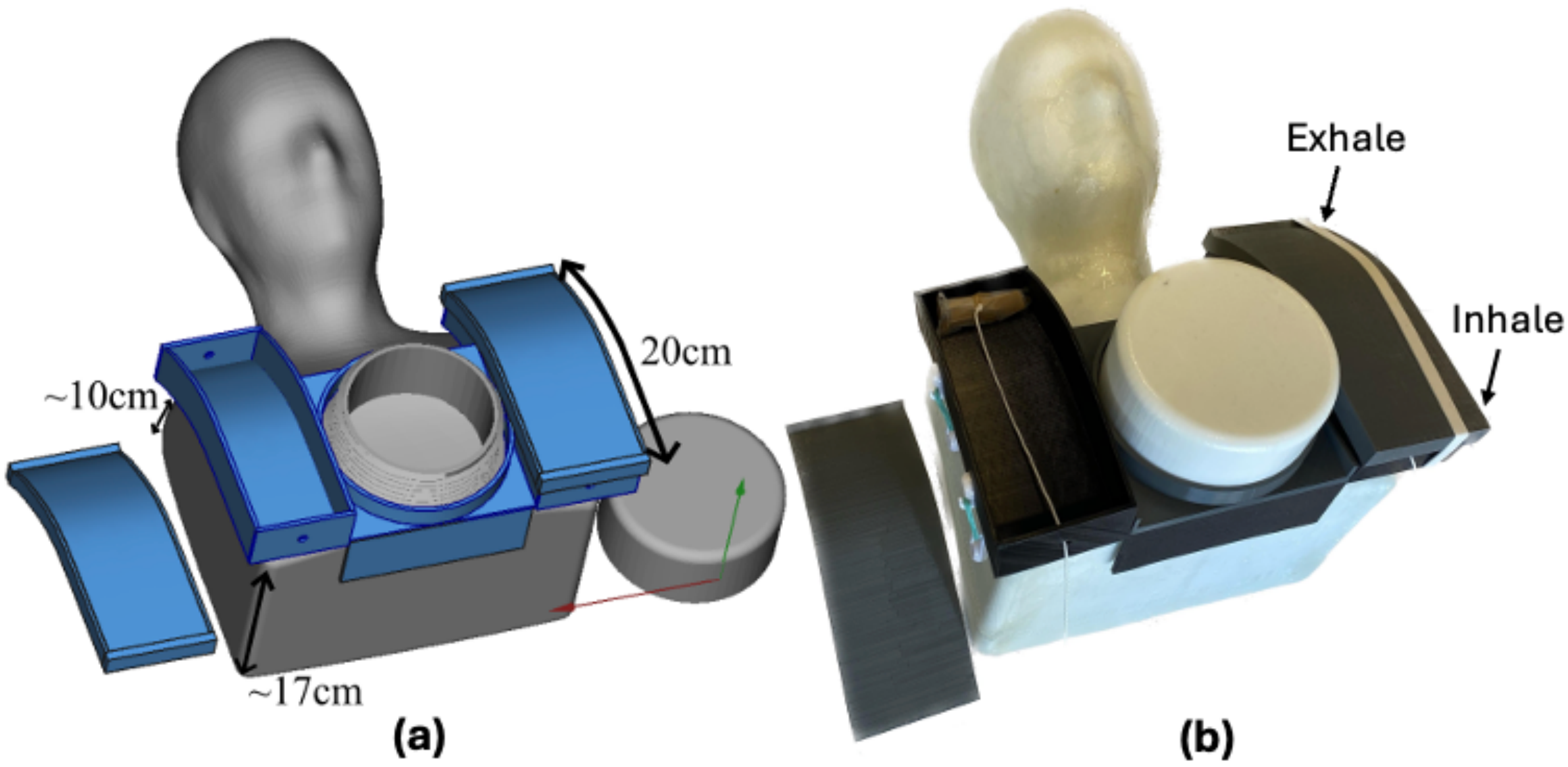

Fig. S3 Digital models (**a**) for the phantom and the respiratory system vest, and corresponding 3D-printed objects (**b**). The vest was designed featuring two distinct enclosed pathways, with removable lids, to be positioned on the phantom thorax. The pathways' length was 20 cm. Iron staples positioned within plastic tubes were moved along each pathway, pulling polyester silk threads attached to the tubes. The periodic movement was used to reproduce breathing-induced field fluctuations in the phantom.

2. Results

2.1 Figures

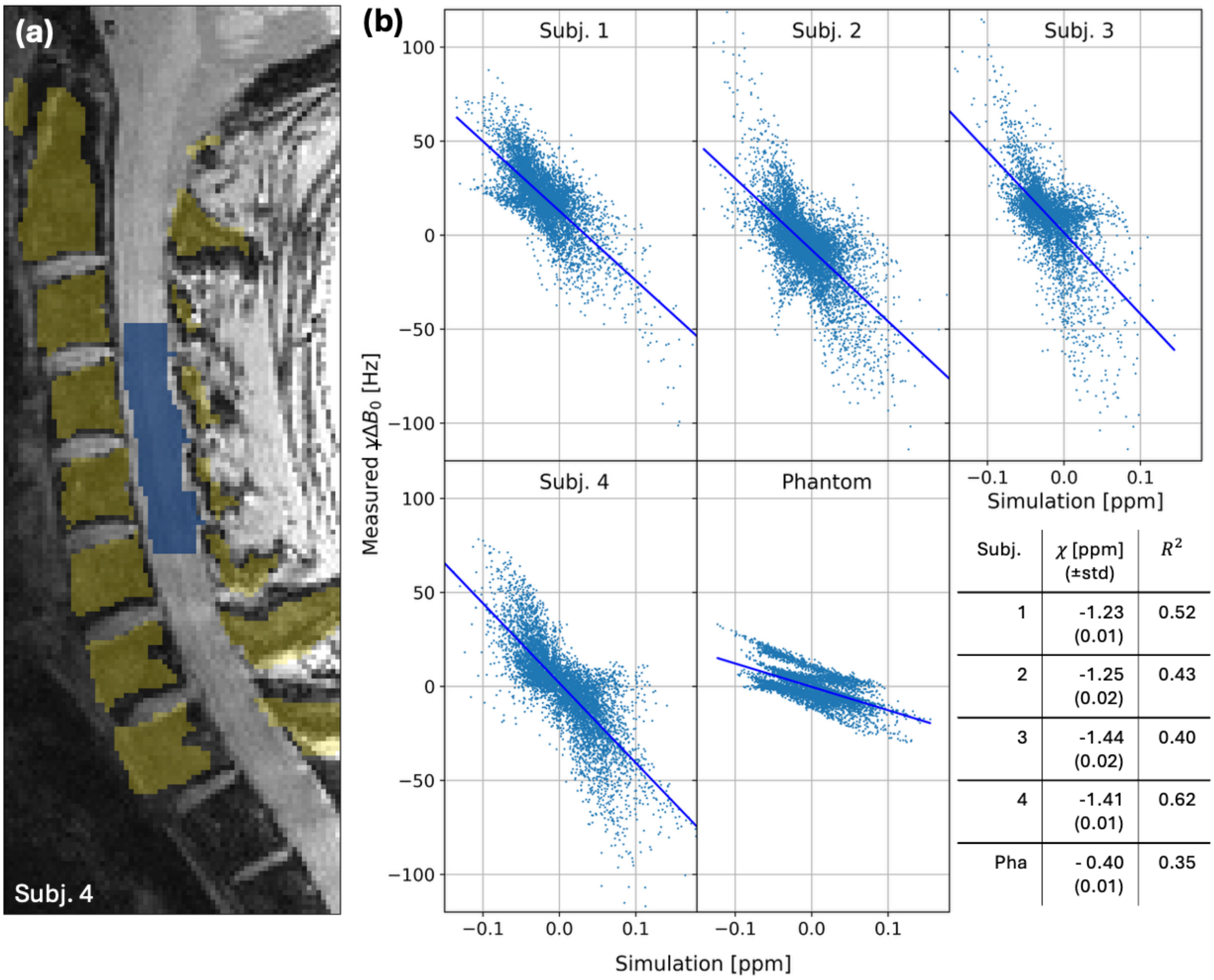

Subj.	χ [ppm] (±std)	R^2
1	-1.23 (0.01)	0.52
2	-1.25 (0.02)	0.43
3	-1.44 (0.02)	0.40
4	-1.41 (0.01)	0.62
Pha	- 0.40 (0.01)	0.35

Fig. S4 A sagittal slice of the second echo of the field map magnitude of subject 4 is displayed in (**a**), overlaid with the registered vertebrae segmentation (in red) and the region of interest for the linear regression (in blue). The results of the linear regression between the measured field fluctuations and the simulations are shown in (**b**), for all subjects and the phantom. The linear regression slope represents an estimate of the susceptibility difference between the vertebrae and the surrounding tissue. The fit residuals are displayed in Figure S5.

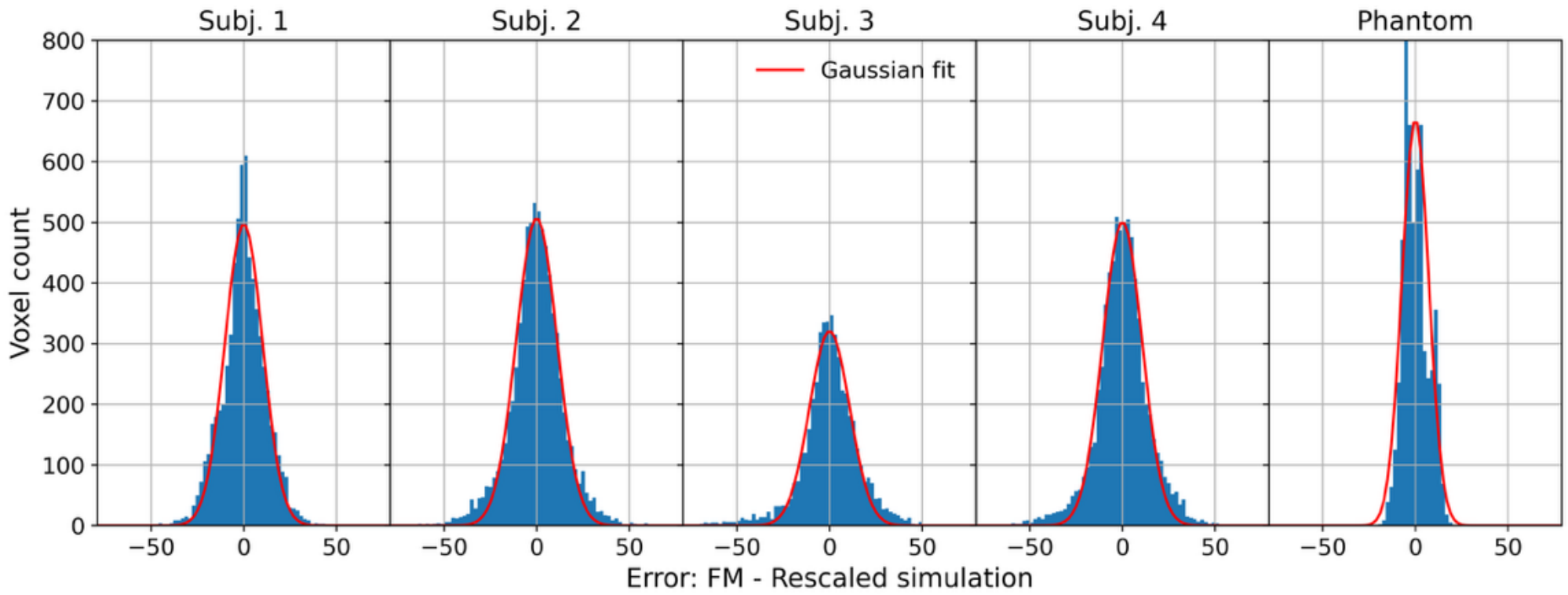

Fig. S5 The fit residuals for the linear regression between the measured local field inhomogeneities and the simulations are shown for all subjects and the phantom. The linear regression was used to obtain an estimate of the susceptibility difference between the vertebrae and the surrounding tissue. The residuals appeared normally distributed, suggesting that the variability is largely attributable to random noise.

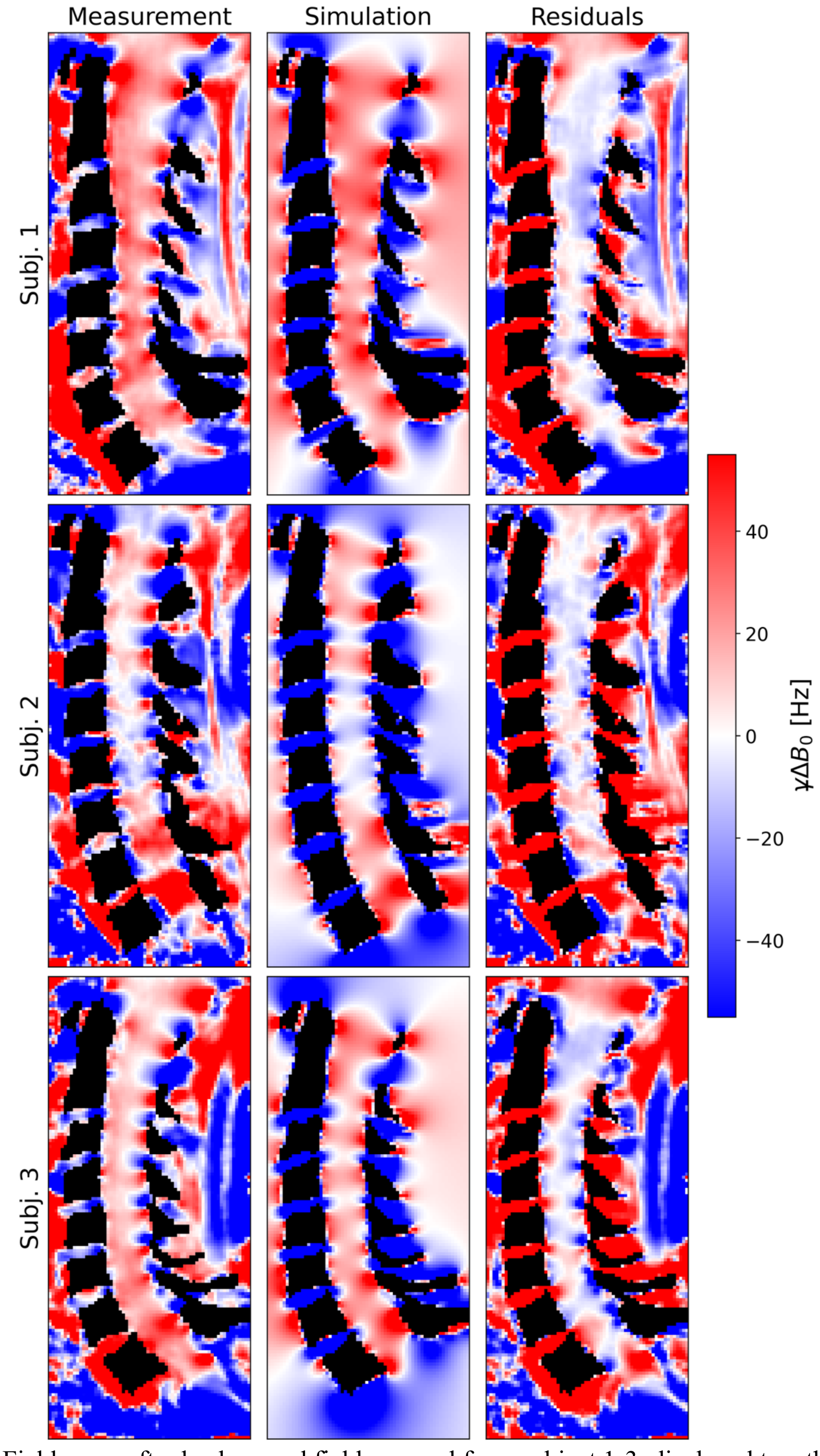

Fig. S6 Field maps after background field removal from subject 1-3, displayed together with the field simulations and their difference (residuals). This figure complements Figure 3 in the main text.

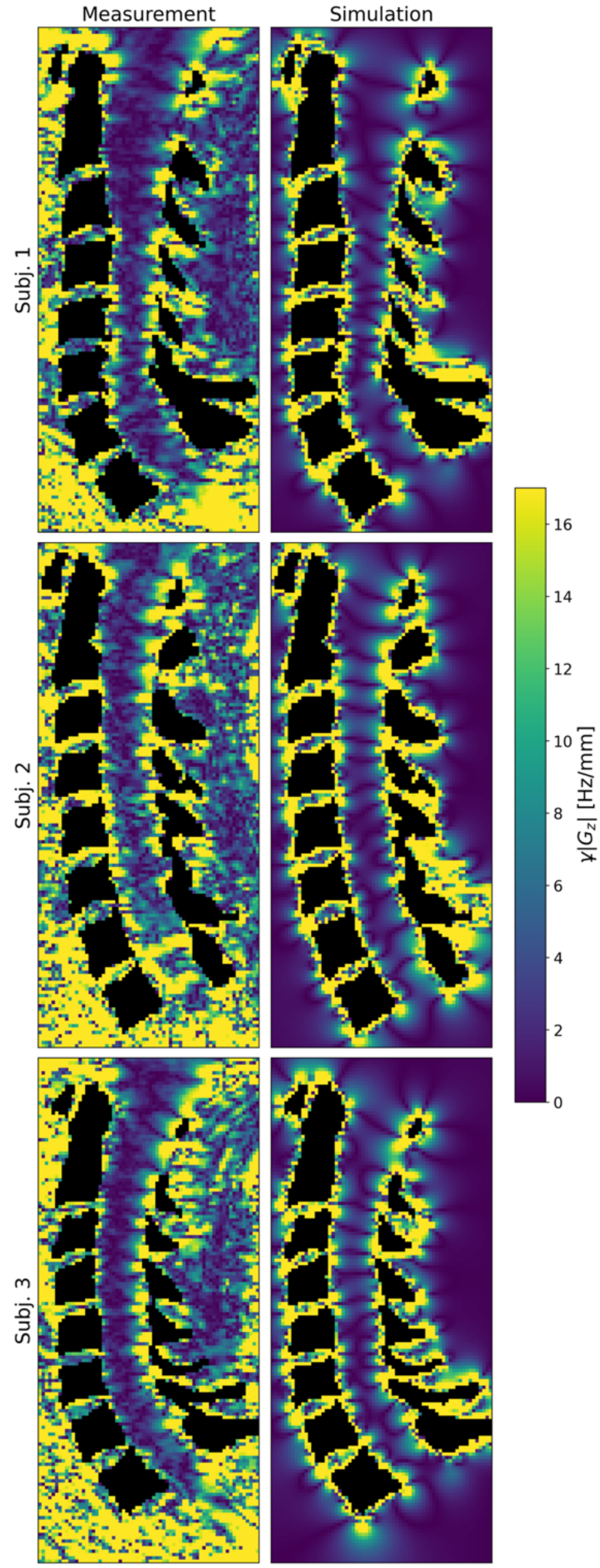

Fig. S7 Gradient maps (z-direction) acquired in subject 1-3 displayed together with the field simulations results. This figure complements Figure 4 in the main text.

3. References

1. Lopez-Rios N, Gilbert KM, Papp D, Cereza G, Foias A, Rangaprakash D, May MW, Guerin B, Wald LL, Keil B, Stockmann JP, Barry RL, Cohen-Adad J (2023) An 8-channel Tx dipole and 20-channel Rx loop coil array for MRI of the cervical spinal cord at 7 Tesla. NMR Biomed 36:e5002.

2. EOS. Safety data sheet PA 2200. https://3dformtech.fi/wp-content/uploads/2019/11/Material-Data-PA2200.pdf.

3. Cyberbond. Safety data sheet 2028 instant adhesive. https://www.eal.com.au/wp-content/uploads/2014/08/CB2028_TDS_AU.pdf.

4. Biltema. Laminating epoxy, 1.5 litre. https://www.biltema.no/en-no/boat/boat-care/epoxy/laminating-epoxy-15-litre-2000052332.

5. Biltema. Glassfibervev (fiberglass fabric), 5 m^2. https://www.biltema.no/batutstyr/batpleie/Glassfiber/glassfibervev-5-m2-2000060474.

6. Massire A, Taso M, Besson P, Guye M, Ranjeva J-P, Callot V (2016) High-resolution multi-parametric quantitative magnetic resonance imaging of the human cervical spinal cord at 7T. NeuroImage 143:58–69.

7. Formlabs. Safety data sheet Rigid 10K. https://center3dprint.com/files/Rigid_10K_Resin_SDS_(EN).pdf.

8. Sangal M, Anikeeva M, Priese SC, Mattern H, Hövener J-B, Speck O (2023) MR based magnetic susceptibility measurements of 3D printing materials at 3 Tesla. J Magn Reson Open 16–17:100138.

9. Dimov AV, Liu Z, Spincemaille P, Prince MR, Du J, Wang Y (2018) Bone quantitative susceptibility mapping using a chemical species–specific signal model with ultrashort and conventional echo data. Magn Reson Med 79:121–128.

10. Buch S, Liu S, Ye Y, Cheng Y-CN, Neelavalli J, Haacke EM (2015) Susceptibility mapping of air, bone, and calcium in the head. Magn Reson Med 73:2185–2194.

11. Hopkins JA, Wehrli FW (1997) Magnetic susceptibility measurement of insoluble solids by NMR: Magnetic susceptibility of bone. Magn Reson Med 37:494–500.

12. de Rochefort L, Brown R, Prince MR, Wang Y (2008) Quantitative MR susceptibility mapping using piece-wise constant regularized inversion of the magnetic field. Magn Reson Med 60:1003–1009.

13. Jula. Betongkrok (concrete hook). https://www.jula.no/catalog/bygg-og-maling/beslag-og-byggematerial/innretningsbeslag/opphengskroker/betongkrok-015366/.

14. Duan Q, Duyn JH, Gudino N, de Zwart JA, van Gelderen P, Sodickson DK, Brown R (2014) Characterization of a dielectric phantom for high-field magnetic resonance imaging applications. Med Phys 41:102303.

15. Änglamark. Liquid hand soap, 500ml (refill bag). https://svanemerket.no/miljomerkede-produkter/produkt/nglamark-liquid-hand-soap-500-ml-refill-bag-110241/.

16. Alonso-Ortiz E, Papp D, D'Astous A, Cohen-Adad J (2023) Dynamic shimming in the cervical spinal cord for multi-echo gradient-echo imaging at 3 T. Neuroimage Rep 3:100150.

17. Kasper L, Bollmann S, Diaconescu AO, Hutton C, Heinzle J, Iglesias S, Hauser TU, Sebold M, Manjaly Z-M, Pruessmann KP, Stephan KE (2017) The PhysIO Toolbox for Modeling Physiological Noise in fMRI Data. J Neurosci Methods 276:56–72.

18. Van Der Walt S, Schönberger JL, Nunez-Iglesias J, Boulogne F, Warner JD, Yager N, Gouillart E, Yu T (2014) scikit-image: image processing in Python. PeerJ 2:e453.

19. FFmpeg Developers (2024) FFmpeg: A complete, cross-platform solution to record, convert and stream audio and video.
